# Search for invisible particles produced in association with single-top-quarks in proton–proton collisions at $\sqrt{s}=\mathrm {8~TeV}$ with the ATLAS detector

**DOI:** 10.1140/epjc/s10052-014-3233-4

**Published:** 2015-02-18

**Authors:** ATLAS Collaboration, G. Aad, B. Abbott, J. Abdallah, S. Abdel Khalek, O. Abdinov, R. Aben, B. Abi, M. Abolins, O. S. AbouZeid, H. Abramowicz, H. Abreu, R. Abreu, Y. Abulaiti, B. S. Acharya, L. Adamczyk, D. L. Adams, J. Adelman, S. Adomeit, T. Adye, T. Agatonovic-Jovin, J. A. Aguilar-Saavedra, M. Agustoni, S. P. Ahlen, F. Ahmadov, G. Aielli, H. Akerstedt, T. P. A. Åkesson, G. Akimoto, A. V. Akimov, G. L. Alberghi, J. Albert, S. Albrand, M. J. Alconada Verzini, M. Aleksa, I. N. Aleksandrov, C. Alexa, G. Alexander, G. Alexandre, T. Alexopoulos, M. Alhroob, G. Alimonti, L. Alio, J. Alison, B. M. M. Allbrooke, L. J. Allison, P. P. Allport, A. Aloisio, A. Alonso, F. Alonso, C. Alpigiani, A. Altheimer, B. Alvarez Gonzalez, M. G. Alviggi, K. Amako, Y. Amaral Coutinho, C. Amelung, D. Amidei, S. P. Amor Dos Santos, A. Amorim, S. Amoroso, N. Amram, G. Amundsen, C. Anastopoulos, L. S. Ancu, N. Andari, T. Andeen, C. F. Anders, G. Anders, K. J. Anderson, A. Andreazza, V. Andrei, X. S. Anduaga, S. Angelidakis, I. Angelozzi, P. Anger, A. Angerami, F. Anghinolfi, A. V. Anisenkov, N. Anjos, A. Annovi, M. Antonelli, A. Antonov, J. Antos, F. Anulli, M. Aoki, L. Aperio Bella, R. Apolle, G. Arabidze, I. Aracena, Y. Arai, J. P. Araque, A. T. H. Arce, F. A. Arduh, J.-F. Arguin, S. Argyropoulos, M. Arik, A. J. Armbruster, O. Arnaez, V. Arnal, H. Arnold, M. Arratia, O. Arslan, A. Artamonov, G. Artoni, S. Asai, N. Asbah, A. Ashkenazi, B. Åsman, L. Asquith, K. Assamagan, R. Astalos, M. Atkinson, N. B. Atlay, B. Auerbach, K. Augsten, M. Aurousseau, G. Avolio, B. Axen, G. Azuelos, Y. Azuma, M. A. Baak, A. E. Baas, C. Bacci, H. Bachacou, K. Bachas, M. Backes, M. Backhaus, E. Badescu, P. Bagiacchi, P. Bagnaia, Y. Bai, T. Bain, J. T. Baines, O. K. Baker, P. Balek, F. Balli, E. Banas, Sw. Banerjee, A. A. E. Bannoura, H. S. Bansil, L. Barak, S. P. Baranov, E. L. Barberio, D. Barberis, M. Barbero, T. Barillari, M. Barisonzi, T. Barklow, N. Barlow, S. L. Barnes, B. M. Barnett, R. M. Barnett, Z. Barnovska, A. Baroncelli, G. Barone, A. J. Barr, F. Barreiro, J. Barreiro Guimarães da Costa, R. Bartoldus, A. E. Barton, P. Bartos, V. Bartsch, A. Bassalat, A. Basye, R. L. Bates, S. J. Batista, J. R. Batley, M. Battaglia, M. Battistin, F. Bauer, H. S. Bawa, J. B. Beacham, M. D. Beattie, T. Beau, P. H. Beauchemin, R. Beccherle, P. Bechtle, H. P. Beck, K. Becker, S. Becker, M. Beckingham, C. Becot, A. J. Beddall, A. Beddall, S. Bedikian, V. A. Bednyakov, C. P. Bee, L. J. Beemster, T. A. Beermann, M. Begel, K. Behr, C. Belanger-Champagne, P. J. Bell, W. H. Bell, G. Bella, L. Bellagamba, A. Bellerive, M. Bellomo, K. Belotskiy, O. Beltramello, O. Benary, D. Benchekroun, K. Bendtz, N. Benekos, Y. Benhammou, E. Benhar Noccioli, J. A. Benitez Garcia, D. P. Benjamin, J. R. Bensinger, S. Bentvelsen, D. Berge, E. Bergeaas Kuutmann, N. Berger, F. Berghaus, J. Beringer, C. Bernard, N. R. Bernard, C. Bernius, F. U. Bernlochner, T. Berry, P. Berta, C. Bertella, G. Bertoli, F. Bertolucci, C. Bertsche, D. Bertsche, M. I. Besana, G. J. Besjes, O. Bessidskaia Bylund, M. Bessner, N. Besson, C. Betancourt, S. Bethke, W. Bhimji, R. M. Bianchi, L. Bianchini, M. Bianco, O. Biebel, S. P. Bieniek, K. Bierwagen, M. Biglietti, J. Bilbao De Mendizabal, H. Bilokon, M. Bindi, S. Binet, A. Bingul, C. Bini, C. W. Black, J. E. Black, K. M. Black, D. Blackburn, R. E. Blair, J.-B. Blanchard, T. Blazek, I. Bloch, C. Blocker, W. Blum, U. Blumenschein, G. J. Bobbink, V. S. Bobrovnikov, S. S. Bocchetta, A. Bocci, C. Bock, C. R. Boddy, M. Boehler, T. T. Boek, J. A. Bogaerts, A. G. Bogdanchikov, A. Bogouch, C. Bohm, V. Boisvert, T. Bold, V. Boldea, A. S. Boldyrev, M. Bomben, M. Bona, M. Boonekamp, A. Borisov, G. Borissov, M. Borri, S. Borroni, J. Bortfeldt, V. Bortolotto, K. Bos, D. Boscherini, M. Bosman, H. Boterenbrood, J. Boudreau, J. Bouffard, E. V. Bouhova-Thacker, D. Boumediene, C. Bourdarios, N. Bousson, S. Boutouil, A. Boveia, J. Boyd, I. R. Boyko, I. Bozic, J. Bracinik, A. Brandt, G. Brandt, O. Brandt, U. Bratzler, B. Brau, J. E. Brau, H. M. Braun, S. F. Brazzale, B. Brelier, K. Brendlinger, A. J. Brennan, R. Brenner, S. Bressler, K. Bristow, T. M. Bristow, D. Britton, F. M. Brochu, I. Brock, R. Brock, J. Bronner, G. Brooijmans, T. Brooks, W. K. Brooks, J. Brosamer, E. Brost, J. Brown, P. A. Bruckman de Renstrom, D. Bruncko, R. Bruneliere, S. Brunet, A. Bruni, G. Bruni, M. Bruschi, L. Bryngemark, T. Buanes, Q. Buat, F. Bucci, P. Buchholz, A. G. Buckley, S. I. Buda, I. A. Budagov, F. Buehrer, L. Bugge, M. K. Bugge, O. Bulekov, A. C. Bundock, H. Burckhart, S. Burdin, B. Burghgrave, S. Burke, I. Burmeister, E. Busato, D. Büscher, V. Büscher, P. Bussey, C. P. Buszello, B. Butler, J. M. Butler, A. I. Butt, C. M. Buttar, J. M. Butterworth, P. Butti, W. Buttinger, A. Buzatu, M. Byszewski, S. Cabrera Urbán, D. Caforio, O. Cakir, P. Calafiura, A. Calandri, G. Calderini, P. Calfayan, L. P. Caloba, D. Calvet, S. Calvet, R. Camacho Toro, S. Camarda, D. Cameron, L. M. Caminada, R. Caminal Armadans, S. Campana, M. Campanelli, A. Campoverde, V. Canale, A. Canepa, M. Cano Bret, J. Cantero, R. Cantrill, T. Cao, M. D. M. Capeans Garrido, I. Caprini, M. Caprini, M. Capua, R. Caputo, R. Cardarelli, T. Carli, G. Carlino, L. Carminati, S. Caron, E. Carquin, G. D. Carrillo-Montoya, J. R. Carter, J. Carvalho, D. Casadei, M. P. Casado, M. Casolino, E. Castaneda-Miranda, A. Castelli, V. Castillo Gimenez, N. F. Castro, P. Catastini, A. Catinaccio, J. R. Catmore, A. Cattai, G. Cattani, J. Caudron, V. Cavaliere, D. Cavalli, M. Cavalli-Sforza, V. Cavasinni, F. Ceradini, B. C. Cerio, K. Cerny, A. S. Cerqueira, A. Cerri, L. Cerrito, F. Cerutti, M. Cerv, A. Cervelli, S. A. Cetin, A. Chafaq, D. Chakraborty, I. Chalupkova, P. Chang, B. Chapleau, J. D. Chapman, D. Charfeddine, D. G. Charlton, C. C. Chau, C. A. Chavez Barajas, S. Cheatham, A. Chegwidden, S. Chekanov, S. V. Chekulaev, G. A. Chelkov, M. A. Chelstowska, C. Chen, H. Chen, K. Chen, L. Chen, S. Chen, X. Chen, Y. Chen, H. C. Cheng, Y. Cheng, A. Cheplakov, E. Cheremushkina, R. Cherkaoui El Moursli, V. Chernyatin, E. Cheu, L. Chevalier, V. Chiarella, G. Chiefari, J. T. Childers, A. Chilingarov, G. Chiodini, A. S. Chisholm, R. T. Chislett, A. Chitan, M. V. Chizhov, S. Chouridou, B. K. B. Chow, D. Chromek-Burckhart, M. L. Chu, J. Chudoba, J. J. Chwastowski, L. Chytka, G. Ciapetti, A. K. Ciftci, R. Ciftci, D. Cinca, V. Cindro, A. Ciocio, Z. H. Citron, M. Citterio, M. Ciubancan, A. Clark, P. J. Clark, R. N. Clarke, W. Cleland, J. C. Clemens, C. Clement, Y. Coadou, M. Cobal, A. Coccaro, J. Cochran, L. Coffey, J. G. Cogan, B. Cole, S. Cole, A. P. Colijn, J. Collot, T. Colombo, G. Compostella, P. Conde Muiño, E. Coniavitis, S. H. Connell, I. A. Connelly, S. M. Consonni, V. Consorti, S. Constantinescu, C. Conta, G. Conti, F. Conventi, M. Cooke, B. D. Cooper, A. M. Cooper-Sarkar, N. J. Cooper-Smith, K. Copic, T. Cornelissen, M. Corradi, F. Corriveau, A. Corso-Radu, A. Cortes-Gonzalez, G. Cortiana, G. Costa, M. J. Costa, D. Costanzo, D. Côté, G. Cottin, G. Cowan, B. E. Cox, K. Cranmer, G. Cree, S. Crépé-Renaudin, F. Crescioli, W. A. Cribbs, M. Crispin Ortuzar, M. Cristinziani, V. Croft, G. Crosetti, T. Cuhadar Donszelmann, J. Cummings, M. Curatolo, C. Cuthbert, H. Czirr, P. Czodrowski, S. D’Auria, M. D’Onofrio, M. J. Da Cunha Sargedas De Sousa, C. Da Via, W. Dabrowski, A. Dafinca, T. Dai, O. Dale, F. Dallaire, C. Dallapiccola, M. Dam, A. C. Daniells, M. Danninger, M. Dano Hoffmann, V. Dao, G. Darbo, S. Darmora, J. Dassoulas, A. Dattagupta, W. Davey, C. David, T. Davidek, E. Davies, M. Davies, O. Davignon, A. R. Davison, P. Davison, Y. Davygora, E. Dawe, I. Dawson, R. K. Daya-Ishmukhametova, K. De, R. de Asmundis, S. De Castro, S. De Cecco, N. De Groot, P. de Jong, H. De la Torre, F. De Lorenzi, L. De Nooij, D. De Pedis, A. De Salvo, U. De Sanctis, A. De Santo, J. B. De Vivie De Regie, W. J. Dearnaley, R. Debbe, C. Debenedetti, B. Dechenaux, D. V. Dedovich, I. Deigaard, J. Del Peso, T. Del Prete, F. Deliot, C. M. Delitzsch, M. Deliyergiyev, A. Dell’Acqua, L. Dell’Asta, M. Dell’Orso, M. Della Pietra, D. della Volpe, M. Delmastro, P. A. Delsart, C. Deluca, D. A. DeMarco, S. Demers, M. Demichev, A. Demilly, S. P. Denisov, D. Derendarz, J. E. Derkaoui, F. Derue, P. Dervan, K. Desch, C. Deterre, P. O. Deviveiros, A. Dewhurst, S. Dhaliwal, A. Di Ciaccio, L. Di Ciaccio, A. Di Domenico, C. Di Donato, A. Di Girolamo, B. Di Girolamo, A. Di Mattia, B. Di Micco, R. Di Nardo, A. Di Simone, R. Di Sipio, D. Di Valentino, F. A. Dias, M. A. Diaz, E. B. Diehl, J. Dietrich, T. A. Dietzsch, S. Diglio, A. Dimitrievska, J. Dingfelder, P. Dita, S. Dita, F. Dittus, F. Djama, T. Djobava, J. I. Djuvsland, M. A. B. do Vale, D. Dobos, C. Doglioni, T. Doherty, T. Dohmae, J. Dolejsi, Z. Dolezal, B. A. Dolgoshein, M. Donadelli, S. Donati, P. Dondero, J. Donini, J. Dopke, A. Doria, M. T. Dova, A. T. Doyle, M. Dris, J. Dubbert, S. Dube, E. Dubreuil, E. Duchovni, G. Duckeck, O. A. Ducu, D. Duda, A. Dudarev, F. Dudziak, L. Duflot, L. Duguid, M. Dührssen, M. Dunford, H. Duran Yildiz, M. Düren, A. Durglishvili, D. Duschinger, M. Dwuznik, M. Dyndal, J. Ebke, W. Edson, N. C. Edwards, W. Ehrenfeld, T. Eifert, G. Eigen, K. Einsweiler, T. Ekelof, M. El Kacimi, M. Ellert, S. Elles, F. Ellinghaus, N. Ellis, J. Elmsheuser, M. Elsing, D. Emeliyanov, Y. Enari, O. C. Endner, M. Endo, R. Engelmann, J. Erdmann, A. Ereditato, D. Eriksson, G. Ernis, J. Ernst, M. Ernst, J. Ernwein, S. Errede, E. Ertel, M. Escalier, H. Esch, C. Escobar, B. Esposito, A. I. Etienvre, E. Etzion, H. Evans, A. Ezhilov, L. Fabbri, G. Facini, R. M. Fakhrutdinov, S. Falciano, R. J. Falla, J. Faltova, Y. Fang, M. Fanti, A. Farbin, A. Farilla, T. Farooque, S. Farrell, S. M. Farrington, P. Farthouat, F. Fassi, P. Fassnacht, D. Fassouliotis, A. Favareto, L. Fayard, P. Federic, O. L. Fedin, W. Fedorko, S. Feigl, L. Feligioni, C. Feng, E. J. Feng, H. Feng, A. B. Fenyuk, P. Fernandez Martinez, S. Fernandez Perez, S. Ferrag, J. Ferrando, A. Ferrari, P. Ferrari, R. Ferrari, D. E. Ferreira de Lima, A. Ferrer, D. Ferrere, C. Ferretti, A. Ferretto Parodi, M. Fiascaris, F. Fiedler, A. Filipčič, M. Filipuzzi, F. Filthaut, M. Fincke-Keeler, K. D. Finelli, M. C. N. Fiolhais, L. Fiorini, A. Firan, A. Fischer, J. Fischer, W. C. Fisher, E. A. Fitzgerald, M. Flechl, I. Fleck, P. Fleischmann, S. Fleischmann, G. T. Fletcher, G. Fletcher, T. Flick, A. Floderus, L. R. Flores Castillo, M. J. Flowerdew, A. Formica, A. Forti, D. Fournier, H. Fox, S. Fracchia, P. Francavilla, M. Franchini, S. Franchino, D. Francis, L. Franconi, M. Franklin, M. Fraternali, S. T. French, C. Friedrich, F. Friedrich, D. Froidevaux, J. A. Frost, C. Fukunaga, E. Fullana Torregrosa, B. G. Fulsom, J. Fuster, C. Gabaldon, O. Gabizon, A. Gabrielli, A. Gabrielli, S. Gadatsch, S. Gadomski, G. Gagliardi, P. Gagnon, C. Galea, B. Galhardo, E. J. Gallas, B. J. Gallop, P. Gallus, G. Galster, K. K. Gan, J. Gao, Y. S. Gao, F. M. Garay Walls, F. Garberson, C. García, J. E. García Navarro, M. Garcia-Sciveres, R. W. Gardner, N. Garelli, V. Garonne, C. Gatti, G. Gaudio, B. Gaur, L. Gauthier, P. Gauzzi, I. L. Gavrilenko, C. Gay, G. Gaycken, E. N. Gazis, P. Ge, Z. Gecse, C. N. P. Gee, D. A. A. Geerts, Ch. Geich-Gimbel, K. Gellerstedt, C. Gemme, A. Gemmell, M. H. Genest, S. Gentile, M. George, S. George, D. Gerbaudo, A. Gershon, H. Ghazlane, N. Ghodbane, B. Giacobbe, S. Giagu, V. Giangiobbe, P. Giannetti, F. Gianotti, B. Gibbard, S. M. Gibson, M. Gilchriese, T. P. S. Gillam, D. Gillberg, G. Gilles, D. M. Gingrich, N. Giokaris, M. P. Giordani, R. Giordano, F. M. Giorgi, F. M. Giorgi, P. F. Giraud, D. Giugni, C. Giuliani, M. Giulini, B. K. Gjelsten, S. Gkaitatzis, I. Gkialas, E. L. Gkougkousis, L. K. Gladilin, C. Glasman, J. Glatzer, P. C. F. Glaysher, A. Glazov, G. L. Glonti, M. Goblirsch-Kolb, J. R. Goddard, J. Godlewski, S. Goldfarb, T. Golling, D. Golubkov, A. Gomes, L. S. Gomez Fajardo, R. Gonçalo, J. Goncalves Pinto Firmino Da Costa, L. Gonella, S. González de la Hoz, G. Gonzalez Parra, S. Gonzalez-Sevilla, L. Goossens, P. A. Gorbounov, H. A. Gordon, I. Gorelov, B. Gorini, E. Gorini, A. Gorišek, E. Gornicki, A. T. Goshaw, C. Gössling, M. I. Gostkin, M. Gouighri, D. Goujdami, M. P. Goulette, A. G. Goussiou, C. Goy, H. M. X. Grabas, L. Graber, I. Grabowska-Bold, P. Grafström, K.-J. Grahn, J. Gramling, E. Gramstad, S. Grancagnolo, V. Grassi, V. Gratchev, H. M. Gray, E. Graziani, O. G. Grebenyuk, Z. D. Greenwood, K. Gregersen, I. M. Gregor, P. Grenier, J. Griffiths, A. A. Grillo, K. Grimm, S. Grinstein, Ph. Gris, Y. V. Grishkevich, J.-F. Grivaz, J. P. Grohs, A. Grohsjean, E. Gross, J. Grosse-Knetter, G. C. Grossi, Z. J. Grout, L. Guan, J. Guenther, F. Guescini, D. Guest, O. Gueta, C. Guicheney, E. Guido, T. Guillemin, S. Guindon, U. Gul, C. Gumpert, J. Guo, S. Gupta, P. Gutierrez, N. G. Gutierrez Ortiz, C. Gutschow, N. Guttman, C. Guyot, C. Gwenlan, C. B. Gwilliam, A. Haas, C. Haber, H. K. Hadavand, N. Haddad, P. Haefner, S. Hageböeck, Z. Hajduk, H. Hakobyan, M. Haleem, D. Hall, G. Halladjian, G. D. Hallewell, K. Hamacher, P. Hamal, K. Hamano, M. Hamer, A. Hamilton, S. Hamilton, G. N. Hamity, P. G. Hamnett, L. Han, K. Hanagaki, K. Hanawa, M. Hance, P. Hanke, R. Hann, J. B. Hansen, J. D. Hansen, P. H. Hansen, K. Hara, A. S. Hard, T. Harenberg, F. Hariri, S. Harkusha, R. D. Harrington, O. M. Harris, P. F. Harrison, F. Hartjes, M. Hasegawa, S. Hasegawa, Y. Hasegawa, A. Hasib, S. Hassani, S. Haug, M. Hauschild, R. Hauser, M. Havranek, C. M. Hawkes, R. J. Hawkings, A. D. Hawkins, T. Hayashi, D. Hayden, C. P. Hays, J. M. Hays, H. S. Hayward, S. J. Haywood, S. J. Head, T. Heck, V. Hedberg, L. Heelan, S. Heim, T. Heim, B. Heinemann, L. Heinrich, J. Hejbal, L. Helary, C. Heller, M. Heller, S. Hellman, D. Hellmich, C. Helsens, J. Henderson, Y. Heng, R. C. W. Henderson, C. Hengler, A. Henrichs, A. M. Henriques Correia, S. Henrot-Versille, G. H. Herbert, Y. Hernández Jiménez, R. Herrberg-Schubert, G. Herten, R. Hertenberger, L. Hervas, G. G. Hesketh, N. P. Hessey, R. Hickling, E. Higón-Rodriguez, E. Hill, J. C. Hill, K. H. Hiller, S. J. Hillier, I. Hinchliffe, E. Hines, M. Hirose, D. Hirschbuehl, J. Hobbs, N. Hod, M. C. Hodgkinson, P. Hodgson, A. Hoecker, M. R. Hoeferkamp, F. Hoenig, D. Hoffmann, M. Hohlfeld, T. R. Holmes, T. M. Hong, L. Hooft van Huysduynen, W. H. Hopkins, Y. Horii, A. J. Horton, J.-Y. Hostachy, S. Hou, A. Hoummada, J. Howard, J. Howarth, M. Hrabovsky, I. Hristova, J. Hrivnac, T. Hryn’ova, A. Hrynevich, C. Hsu, P. J. Hsu, S.-C. Hsu, D. Hu, X. Hu, Y. Huang, Z. Hubacek, F. Hubaut, F. Huegging, T. B. Huffman, E. W. Hughes, G. Hughes, M. Huhtinen, T. A. Hülsing, M. Hurwitz, N. Huseynov, J. Huston, J. Huth, G. Iacobucci, G. Iakovidis, I. Ibragimov, L. Iconomidou-Fayard, E. Ideal, Z. Idrissi, P. Iengo, O. Igonkina, T. Iizawa, Y. Ikegami, K. Ikematsu, M. Ikeno, Y. Ilchenko, D. Iliadis, N. Ilic, Y. Inamaru, T. Ince, P. Ioannou, M. Iodice, K. Iordanidou, V. Ippolito, A. Irles Quiles, C. Isaksson, M. Ishino, M. Ishitsuka, R. Ishmukhametov, C. Issever, S. Istin, J. M. Iturbe Ponce, R. Iuppa, J. Ivarsson, W. Iwanski, H. Iwasaki, J. M. Izen, V. Izzo, B. Jackson, M. Jackson, P. Jackson, M. R. Jaekel, V. Jain, K. Jakobs, S. Jakobsen, T. Jakoubek, J. Jakubek, D. O. Jamin, D. K. Jana, E. Jansen, H. Jansen, J. Janssen, M. Janus, G. Jarlskog, N. Javadov, T. Javůrek, L. Jeanty, J. Jejelava, G.-Y. Jeng, D. Jennens, P. Jenni, J. Jentzsch, C. Jeske, S. Jézéquel, H. Ji, J. Jia, Y. Jiang, M. Jimenez Belenguer, S. Jin, A. Jinaru, O. Jinnouchi, M. D. Joergensen, K. E. Johansson, P. Johansson, K. A. Johns, K. Jon-And, G. Jones, R. W. L. Jones, T. J. Jones, J. Jongmanns, P. M. Jorge, K. D. Joshi, J. Jovicevic, X. Ju, C. A. Jung, P. Jussel, A. Juste Rozas, M. Kaci, A. Kaczmarska, M. Kado, H. Kagan, M. Kagan, E. Kajomovitz, C. W. Kalderon, S. Kama, A. Kamenshchikov, N. Kanaya, M. Kaneda, S. Kaneti, V. A. Kantserov, J. Kanzaki, B. Kaplan, A. Kapliy, D. Kar, K. Karakostas, A. Karamaoun, N. Karastathis, M. J. Kareem, M. Karnevskiy, S. N. Karpov, Z. M. Karpova, K. Karthik, V. Kartvelishvili, A. N. Karyukhin, L. Kashif, G. Kasieczka, R. D. Kass, A. Kastanas, Y. Kataoka, A. Katre, J. Katzy, V. Kaushik, K. Kawagoe, T. Kawamoto, G. Kawamura, S. Kazama, V. F. Kazanin, M. Y. Kazarinov, R. Keeler, R. Kehoe, M. Keil, J. S. Keller, J. J. Kempster, H. Keoshkerian, O. Kepka, B. P. Kerševan, S. Kersten, K. Kessoku, J. Keung, R. A. Keyes, F. Khalil-zada, H. Khandanyan, A. Khanov, A. Kharlamov, A. Khodinov, A. Khomich, T. J. Khoo, G. Khoriauli, V. Khovanskiy, E. Khramov, J. Khubua, H. Y. Kim, H. Kim, S. H. Kim, N. Kimura, O. Kind, B. T. King, M. King, R. S. B. King, S. B. King, J. Kirk, A. E. Kiryunin, T. Kishimoto, D. Kisielewska, F. Kiss, K. Kiuchi, E. Kladiva, M. Klein, U. Klein, K. Kleinknecht, P. Klimek, A. Klimentov, R. Klingenberg, J. A. Klinger, T. Klioutchnikova, P. F. Klok, E.-E. Kluge, P. Kluit, S. Kluth, E. Kneringer, E. B. F. G. Knoops, A. Knue, D. Kobayashi, T. Kobayashi, M. Kobel, M. Kocian, P. Kodys, T. Koffas, E. Koffeman, L. A. Kogan, S. Kohlmann, Z. Kohout, T. Kohriki, T. Koi, H. Kolanoski, I. Koletsou, J. Koll, A. A. Komar, Y. Komori, T. Kondo, N. Kondrashova, K. Köneke, A. C. König, S. König, T. Kono, R. Konoplich, N. Konstantinidis, R. Kopeliansky, S. Koperny, L. Köpke, A. K. Kopp, K. Korcyl, K. Kordas, A. Korn, A. A. Korol, I. Korolkov, E. V. Korolkova, V. A. Korotkov, O. Kortner, S. Kortner, V. V. Kostyukhin, V. M. Kotov, A. Kotwal, A. Kourkoumeli-Charalampidi, C. Kourkoumelis, V. Kouskoura, A. Koutsman, R. Kowalewski, T. Z. Kowalski, W. Kozanecki, A. S. Kozhin, V. A. Kramarenko, G. Kramberger, D. Krasnopevtsev, M. W. Krasny, A. Krasznahorkay, J. K. Kraus, A. Kravchenko, S. Kreiss, M. Kretz, J. Kretzschmar, K. Kreutzfeldt, P. Krieger, K. Kroeninger, H. Kroha, J. Kroll, J. Kroseberg, J. Krstic, U. Kruchonak, H. Krüger, N. Krumnack, Z. V. Krumshteyn, A. Kruse, M. C. Kruse, M. Kruskal, T. Kubota, H. Kucuk, S. Kuday, S. Kuehn, A. Kugel, F. Kuger, A. Kuhl, T. Kuhl, V. Kukhtin, Y. Kulchitsky, S. Kuleshov, M. Kuna, T. Kunigo, A. Kupco, H. Kurashige, Y. A. Kurochkin, R. Kurumida, V. Kus, E. S. Kuwertz, M. Kuze, J. Kvita, D. Kyriazopoulos, A. La Rosa, L. La Rotonda, C. Lacasta, F. Lacava, J. Lacey, H. Lacker, D. Lacour, V. R. Lacuesta, E. Ladygin, R. Lafaye, B. Laforge, T. Lagouri, S. Lai, H. Laier, L. Lambourne, S. Lammers, C. L. Lampen, W. Lampl, E. Lançon, U. Landgraf, M. P. J. Landon, V. S. Lang, A. J. Lankford, F. Lanni, K. Lantzsch, S. Laplace, C. Lapoire, J. F. Laporte, T. Lari, F. Lasagni Manghi, M. Lassnig, P. Laurelli, W. Lavrijsen, A. T. Law, P. Laycock, O. Le Dortz, E. Le Guirriec, E. Le Menedeu, T. LeCompte, F. Ledroit-Guillon, C. A. Lee, H. Lee, S. C. Lee, L. Lee, G. Lefebvre, M. Lefebvre, F. Legger, C. Leggett, A. Lehan, G. Lehmann Miotto, X. Lei, W. A. Leight, A. Leisos, A. G. Leister, M. A. L. Leite, R. Leitner, D. Lellouch, B. Lemmer, K. J. C. Leney, T. Lenz, G. Lenzen, B. Lenzi, R. Leone, S. Leone, C. Leonidopoulos, S. Leontsinis, C. Leroy, C. G. Lester, C. M. Lester, M. Levchenko, J. Levêque, D. Levin, L. J. Levinson, M. Levy, A. Lewis, G. H. Lewis, A. M. Leyko, M. Leyton, B. Li, B. Li, H. Li, H. L. Li, L. Li, L. Li, S. Li, Y. Li, Z. Liang, H. Liao, B. Liberti, P. Lichard, K. Lie, J. Liebal, W. Liebig, C. Limbach, A. Limosani, S. C. Lin, T. H. Lin, F. Linde, B. E. Lindquist, J. T. Linnemann, E. Lipeles, A. Lipniacka, M. Lisovyi, T. M. Liss, D. Lissauer, A. Lister, A. M. Litke, B. Liu, D. Liu, J. B. Liu, K. Liu, L. Liu, M. Liu, M. Liu, Y. Liu, M. Livan, A. Lleres, J. Llorente Merino, S. L. Lloyd, F. Lo Sterzo, E. Lobodzinska, P. Loch, W. S. Lockman, F. K. Loebinger, A. E. Loevschall-Jensen, A. Loginov, T. Lohse, K. Lohwasser, M. Lokajicek, V. P. Lombardo, B. A. Long, J. D. Long, R. E. Long, K. A. Looper, L. Lopes, D. Lopez Mateos, B. Lopez Paredes, I. Lopez Paz, J. Lorenz, N. Lorenzo Martinez, M. Losada, P. Loscutoff, X. Lou, A. Lounis, J. Love, P. A. Love, A. J. Lowe, F. Lu, N. Lu, H. J. Lubatti, C. Luci, A. Lucotte, F. Luehring, W. Lukas, L. Luminari, O. Lundberg, B. Lund-Jensen, M. Lungwitz, D. Lynn, R. Lysak, E. Lytken, H. Ma, L. L. Ma, G. Maccarrone, A. Macchiolo, J. Machado Miguens, D. Macina, D. Madaffari, R. Madar, H. J. Maddocks, W. F. Mader, A. Madsen, M. Maeno, T. Maeno, A. Maevskiy, E. Magradze, K. Mahboubi, J. Mahlstedt, S. Mahmoud, C. Maiani, C. Maidantchik, A. A. Maier, A. Maio, S. Majewski, Y. Makida, N. Makovec, P. Mal, B. Malaescu, Pa. Malecki, V. P. Maleev, F. Malek, U. Mallik, D. Malon, C. Malone, S. Maltezos, V. M. Malyshev, S. Malyukov, J. Mamuzic, B. Mandelli, L. Mandelli, I. Mandić, R. Mandrysch, J. Maneira, A. Manfredini, L. Manhaes de Andrade Filho, J. A. Manjarres Ramos, A. Mann, P. M. Manning, A. Manousakis-Katsikakis, B. Mansoulie, R. Mantifel, M. Mantoani, L. Mapelli, L. March, J. F. Marchand, G. Marchiori, M. Marcisovsky, C. P. Marino, M. Marjanovic, F. Marroquim, S. P. Marsden, Z. Marshall, L. F. Marti, S. Marti-Garcia, B. Martin, B. Martin, T. A. Martin, V. J. Martin, B. Martin dit Latour, H. Martinez, M. Martinez, S. Martin-Haugh, A. C. Martyniuk, M. Marx, F. Marzano, A. Marzin, L. Masetti, T. Mashimo, R. Mashinistov, J. Masik, A. L. Maslennikov, I. Massa, L. Massa, N. Massol, P. Mastrandrea, A. Mastroberardino, T. Masubuchi, P. Mättig, J. Mattmann, J. Maurer, S. J. Maxfield, D. A. Maximov, R. Mazini, L. Mazzaferro, G. Mc Goldrick, S. P. Mc Kee, A. McCarn, R. L. McCarthy, T. G. McCarthy, N. A. McCubbin, K. W. McFarlane, J. A. Mcfayden, G. Mchedlidze, S. J. McMahon, R. A. McPherson, J. Mechnich, M. Medinnis, S. Meehan, S. Mehlhase, A. Mehta, K. Meier, C. Meineck, B. Meirose, C. Melachrinos, B. R. Mellado Garcia, F. Meloni, A. Mengarelli, S. Menke, E. Meoni, K. M. Mercurio, S. Mergelmeyer, N. Meric, P. Mermod, L. Merola, C. Meroni, F. S. Merritt, H. Merritt, A. Messina, J. Metcalfe, A. S. Mete, C. Meyer, C. Meyer, J.-P. Meyer, J. Meyer, R. P. Middleton, S. Migas, S. Miglioranzi, L. Mijović, G. Mikenberg, M. Mikestikova, M. Mikuž, A. Milic, D. W. Miller, C. Mills, A. Milov, D. A. Milstead, A. A. Minaenko, Y. Minami, I. A. Minashvili, A. I. Mincer, B. Mindur, M. Mineev, Y. Ming, L. M. Mir, G. Mirabelli, T. Mitani, J. Mitrevski, V. A. Mitsou, A. Miucci, P. S. Miyagawa, J. U. Mjörnmark, T. Moa, K. Mochizuki, S. Mohapatra, W. Mohr, S. Molander, R. Moles-Valls, K. Mönig, C. Monini, J. Monk, E. Monnier, J. Montejo Berlingen, F. Monticelli, S. Monzani, R. W. Moore, N. Morange, D. Moreno, M. Moreno Llácer, P. Morettini, M. Morgenstern, M. Morii, V. Morisbak, S. Moritz, A. K. Morley, G. Mornacchi, J. D. Morris, A. Morton, L. Morvaj, H. G. Moser, M. Mosidze, J. Moss, K. Motohashi, R. Mount, E. Mountricha, S. V. Mouraviev, E. J. W. Moyse, S. Muanza, R. D. Mudd, F. Mueller, J. Mueller, K. Mueller, T. Mueller, T. Mueller, D. Muenstermann, Y. Munwes, J. A. Murillo Quijada, W. J. Murray, H. Musheghyan, E. Musto, A. G. Myagkov, M. Myska, O. Nackenhorst, J. Nadal, K. Nagai, R. Nagai, Y. Nagai, K. Nagano, A. Nagarkar, Y. Nagasaka, K. Nagata, M. Nagel, A. M. Nairz, Y. Nakahama, K. Nakamura, T. Nakamura, I. Nakano, H. Namasivayam, G. Nanava, R. F. Naranjo Garcia, R. Narayan, T. Nattermann, T. Naumann, G. Navarro, R. Nayyar, H. A. Neal, P. Yu. Nechaeva, T. J. Neep, P. D. Nef, A. Negri, G. Negri, M. Negrini, S. Nektarijevic, C. Nellist, A. Nelson, T. K. Nelson, S. Nemecek, P. Nemethy, A. A. Nepomuceno, M. Nessi, M. S. Neubauer, M. Neumann, R. M. Neves, P. Nevski, P. R. Newman, D. H. Nguyen, R. B. Nickerson, R. Nicolaidou, B. Nicquevert, J. Nielsen, N. Nikiforou, A. Nikiforov, V. Nikolaenko, I. Nikolic-Audit, K. Nikolics, K. Nikolopoulos, P. Nilsson, Y. Ninomiya, A. Nisati, R. Nisius, T. Nobe, M. Nomachi, I. Nomidis, S. Norberg, M. Nordberg, O. Novgorodova, S. Nowak, M. Nozaki, L. Nozka, K. Ntekas, G. Nunes Hanninger, T. Nunnemann, E. Nurse, F. Nuti, B. J. O’Brien, F. O’grady, D. C. O’Neil, V. O’Shea, F. G. Oakham, H. Oberlack, T. Obermann, J. Ocariz, A. Ochi, M. I. Ochoa, S. Oda, S. Odaka, H. Ogren, A. Oh, S. H. Oh, C. C. Ohm, H. Ohman, H. Oide, W. Okamura, H. Okawa, Y. Okumura, T. Okuyama, A. Olariu, A. G. Olchevski, S. A. Olivares Pino, D. Oliveira Damazio, E. Oliver Garcia, A. Olszewski, J. Olszowska, A. Onofre, P. U. E. Onyisi, C. J. Oram, M. J. Oreglia, Y. Oren, D. Orestano, N. Orlando, C. Oropeza Barrera, R. S. Orr, B. Osculati, R. Ospanov, G. Otero y Garzon, H. Otono, M. Ouchrif, E. A. Ouellette, F. Ould-Saada, A. Ouraou, K. P. Oussoren, Q. Ouyang, A. Ovcharova, M. Owen, V. E. Ozcan, N. Ozturk, K. Pachal, A. Pacheco Pages, C. Padilla Aranda, M. Pagáčová, S. Pagan Griso, E. Paganis, C. Pahl, F. Paige, P. Pais, K. Pajchel, G. Palacino, S. Palestini, M. Palka, D. Pallin, A. Palma, J. D. Palmer, Y. B. Pan, E. Panagiotopoulou, J. G. Panduro Vazquez, P. Pani, N. Panikashvili, S. Panitkin, D. Pantea, L. Paolozzi, Th. D. Papadopoulou, K. Papageorgiou, A. Paramonov, D. Paredes Hernandez, M. A. Parker, F. Parodi, J. A. Parsons, U. Parzefall, E. Pasqualucci, S. Passaggio, A. Passeri, F. Pastore, Fr. Pastore, G. Pásztor, S. Pataraia, N. D. Patel, J. R. Pater, S. Patricelli, T. Pauly, J. Pearce, L. E. Pedersen, M. Pedersen, S. Pedraza Lopez, R. Pedro, S. V. Peleganchuk, D. Pelikan, H. Peng, B. Penning, J. Penwell, D. V. Perepelitsa, E. Perez Codina, M. T. Pérez García-Estañ, L. Perini, H. Pernegger, S. Perrella, R. Peschke, V. D. Peshekhonov, K. Peters, R. F. Y. Peters, B. A. Petersen, T. C. Petersen, E. Petit, A. Petridis, C. Petridou, E. Petrolo, F. Petrucci, N. E. Pettersson, R. Pezoa, P. W. Phillips, G. Piacquadio, E. Pianori, A. Picazio, E. Piccaro, M. Piccinini, M. A. Pickering, R. Piegaia, D. T. Pignotti, J. E. Pilcher, A. D. Pilkington, J. Pina, M. Pinamonti, A. Pinder, J. L. Pinfold, A. Pingel, B. Pinto, S. Pires, M. Pitt, C. Pizio, L. Plazak, M.-A. Pleier, V. Pleskot, E. Plotnikova, P. Plucinski, D. Pluth, S. Poddar, F. Podlyski, R. Poettgen, L. Poggioli, D. Pohl, M. Pohl, G. Polesello, A. Policicchio, R. Polifka, A. Polini, C. S. Pollard, V. Polychronakos, K. Pommès, L. Pontecorvo, B. G. Pope, G. A. Popeneciu, D. S. Popovic, A. Poppleton, S. Pospisil, K. Potamianos, I. N. Potrap, C. J. Potter, C. T. Potter, G. Poulard, J. Poveda, V. Pozdnyakov, P. Pralavorio, A. Pranko, S. Prasad, S. Prell, D. Price, J. Price, L. E. Price, D. Prieur, M. Primavera, S. Prince, M. Proissl, K. Prokofiev, F. Prokoshin, E. Protopapadaki, S. Protopopescu, J. Proudfoot, M. Przybycien, H. Przysiezniak, E. Ptacek, D. Puddu, E. Pueschel, D. Puldon, M. Purohit, P. Puzo, J. Qian, G. Qin, Y. Qin, A. Quadt, D. R. Quarrie, W. B. Quayle, M. Queitsch-Maitland, D. Quilty, A. Qureshi, V. Radeka, V. Radescu, S. K. Radhakrishnan, P. Radloff, P. Rados, F. Ragusa, G. Rahal, S. Rajagopalan, M. Rammensee, C. Rangel-Smith, K. Rao, F. Rauscher, T. C. Rave, T. Ravenscroft, M. Raymond, A. L. Read, N. P. Readioff, D. M. Rebuzzi, A. Redelbach, G. Redlinger, R. Reece, K. Reeves, L. Rehnisch, H. Reisin, M. Relich, C. Rembser, H. Ren, Z. L. Ren, A. Renaud, M. Rescigno, S. Resconi, O. L. Rezanova, P. Reznicek, R. Rezvani, R. Richter, M. Ridel, P. Rieck, J. Rieger, M. Rijssenbeek, A. Rimoldi, L. Rinaldi, E. Ritsch, I. Riu, F. Rizatdinova, E. Rizvi, S. H. Robertson, A. Robichaud-Veronneau, D. Robinson, J. E. M. Robinson, A. Robson, C. Roda, L. Rodrigues, S. Roe, O. Røhne, S. Rolli, A. Romaniouk, M. Romano, E. Romero Adam, N. Rompotis, M. Ronzani, L. Roos, E. Ros, S. Rosati, K. Rosbach, M. Rose, P. Rose, P. L. Rosendahl, O. Rosenthal, V. Rossetti, E. Rossi, L. P. Rossi, R. Rosten, M. Rotaru, I. Roth, J. Rothberg, D. Rousseau, C. R. Royon, A. Rozanov, Y. Rozen, X. Ruan, F. Rubbo, I. Rubinskiy, V. I. Rud, C. Rudolph, M. S. Rudolph, F. Rühr, A. Ruiz-Martinez, Z. Rurikova, N. A. Rusakovich, A. Ruschke, H. L. Russell, J. P. Rutherfoord, N. Ruthmann, Y. F. Ryabov, M. Rybar, G. Rybkin, N. C. Ryder, A. F. Saavedra, G. Sabato, S. Sacerdoti, A. Saddique, I. Sadeh, H. F.-W. Sadrozinski, R. Sadykov, F. Safai Tehrani, H. Sakamoto, Y. Sakurai, G. Salamanna, A. Salamon, M. Saleem, D. Salek, P. H. Sales De Bruin, D. Salihagic, A. Salnikov, J. Salt, D. Salvatore, F. Salvatore, A. Salvucci, A. Salzburger, D. Sampsonidis, A. Sanchez, J. Sánchez, V. Sanchez Martinez, H. Sandaker, R. L. Sandbach, H. G. Sander, M. P. Sanders, M. Sandhoff, T. Sandoval, C. Sandoval, R. Sandstroem, D. P. C. Sankey, A. Sansoni, C. Santoni, R. Santonico, H. Santos, I. Santoyo Castillo, K. Sapp, A. Sapronov, J. G. Saraiva, B. Sarrazin, G. Sartisohn, O. Sasaki, Y. Sasaki, G. Sauvage, E. Sauvan, P. Savard, D. O. Savu, C. Sawyer, L. Sawyer, D. H. Saxon, J. Saxon, C. Sbarra, A. Sbrizzi, T. Scanlon, D. A. Scannicchio, M. Scarcella, V. Scarfone, J. Schaarschmidt, P. Schacht, D. Schaefer, R. Schaefer, S. Schaepe, S. Schaetzel, U. Schäfer, A. C. Schaffer, D. Schaile, R. D. Schamberger, V. Scharf, V. A. Schegelsky, D. Scheirich, M. Schernau, M. I. Scherzer, C. Schiavi, J. Schieck, C. Schillo, M. Schioppa, S. Schlenker, E. Schmidt, K. Schmieden, C. Schmitt, S. Schmitt, B. Schneider, Y. J. Schnellbach, U. Schnoor, L. Schoeffel, A. Schoening, B. D. Schoenrock, A. L. S. Schorlemmer, M. Schott, D. Schouten, J. Schovancova, S. Schramm, M. Schreyer, C. Schroeder, N. Schuh, M. J. Schultens, H.-C. Schultz-Coulon, H. Schulz, M. Schumacher, B. A. Schumm, Ph. Schune, C. Schwanenberger, A. Schwartzman, T. A. Schwarz, Ph. Schwegler, Ph. Schwemling, R. Schwienhorst, J. Schwindling, T. Schwindt, M. Schwoerer, F. G. Sciacca, E. Scifo, G. Sciolla, F. Scuri, F. Scutti, J. Searcy, G. Sedov, E. Sedykh, P. Seema, S. C. Seidel, A. Seiden, F. Seifert, J. M. Seixas, G. Sekhniaidze, S. J. Sekula, K. E. Selbach, D. M. Seliverstov, G. Sellers, N. Semprini-Cesari, C. Serfon, L. Serin, L. Serkin, T. Serre, R. Seuster, H. Severini, T. Sfiligoj, F. Sforza, A. Sfyrla, E. Shabalina, M. Shamim, L. Y. Shan, R. Shang, J. T. Shank, M. Shapiro, P. B. Shatalov, K. Shaw, A. Shcherbakova, C. Y. Shehu, P. Sherwood, L. Shi, S. Shimizu, C. O. Shimmin, M. Shimojima, M. Shiyakova, A. Shmeleva, D. Shoaleh Saadi, M. J. Shochet, S. Shojaii, D. Short, S. Shrestha, E. Shulga, M. A. Shupe, S. Shushkevich, P. Sicho, O. Sidiropoulou, D. Sidorov, A. Sidoti, F. Siegert, Dj. Sijacki, J. Silva, Y. Silver, D. Silverstein, S. B. Silverstein, V. Simak, O. Simard, Lj. Simic, S. Simion, E. Simioni, B. Simmons, D. Simon, R. Simoniello, P. Sinervo, N. B. Sinev, G. Siragusa, A. Sircar, A. N. Sisakyan, S. Yu. Sivoklokov, J. Sjölin, T. B. Sjursen, H. P. Skottowe, P. Skubic, M. Slater, T. Slavicek, M. Slawinska, K. Sliwa, V. Smakhtin, B. H. Smart, L. Smestad, S. Yu. Smirnov, Y. Smirnov, L. N. Smirnova, O. Smirnova, K. M. Smith, M. Smizanska, K. Smolek, A. A. Snesarev, G. Snidero, S. Snyder, R. Sobie, F. Socher, A. Soffer, D. A. Soh, C. A. Solans, M. Solar, J. Solc, E. Yu. Soldatov, U. Soldevila, A. A. Solodkov, A. Soloshenko, O. V. Solovyanov, V. Solovyev, P. Sommer, H. Y. Song, N. Soni, A. Sood, A. Sopczak, B. Sopko, V. Sopko, V. Sorin, M. Sosebee, R. Soualah, P. Soueid, A. M. Soukharev, D. South, S. Spagnolo, F. Spanò, W. R. Spearman, F. Spettel, R. Spighi, G. Spigo, L. A. Spiller, M. Spousta, T. Spreitzer, R. D. St. Denis, S. Staerz, J. Stahlman, R. Stamen, S. Stamm, E. Stanecka, C. Stanescu, M. Stanescu-Bellu, M. M. Stanitzki, S. Stapnes, E. A. Starchenko, J. Stark, P. Staroba, P. Starovoitov, R. Staszewski, P. Stavina, P. Steinberg, B. Stelzer, H. J. Stelzer, O. Stelzer-Chilton, H. Stenzel, S. Stern, G. A. Stewart, J. A. Stillings, M. C. Stockton, M. Stoebe, G. Stoicea, P. Stolte, S. Stonjek, A. R. Stradling, A. Straessner, M. E. Stramaglia, J. Strandberg, S. Strandberg, A. Strandlie, E. Strauss, M. Strauss, P. Strizenec, R. Ströhmer, D. M. Strom, R. Stroynowski, A. Strubig, S. A. Stucci, B. Stugu, N. A. Styles, D. Su, J. Su, R. Subramaniam, A. Succurro, Y. Sugaya, C. Suhr, M. Suk, V. V. Sulin, S. Sultansoy, T. Sumida, S. Sun, X. Sun, J. E. Sundermann, K. Suruliz, G. Susinno, M. R. Sutton, Y. Suzuki, M. Svatos, S. Swedish, M. Swiatlowski, I. Sykora, T. Sykora, D. Ta, C. Taccini, K. Tackmann, J. Taenzer, A. Taffard, R. Tafirout, N. Taiblum, H. Takai, R. Takashima, H. Takeda, T. Takeshita, Y. Takubo, M. Talby, A. A. Talyshev, J. Y. C. Tam, K. G. Tan, J. Tanaka, R. Tanaka, S. Tanaka, S. Tanaka, A. J. Tanasijczuk, B. B. Tannenwald, N. Tannoury, S. Tapprogge, S. Tarem, F. Tarrade, G. F. Tartarelli, P. Tas, M. Tasevsky, T. Tashiro, E. Tassi, A. Tavares Delgado, Y. Tayalati, F. E. Taylor, G. N. Taylor, W. Taylor, F. A. Teischinger, M. Teixeira Dias Castanheira, P. Teixeira-Dias, K. K. Temming, H. Ten Kate, P. K. Teng, J. J. Teoh, S. Terada, K. Terashi, J. Terron, S. Terzo, M. Testa, R. J. Teuscher, J. Therhaag, T. Theveneaux-Pelzer, J. P. Thomas, J. Thomas-Wilsker, E. N. Thompson, P. D. Thompson, R. J. Thompson, A. S. Thompson, L. A. Thomsen, E. Thomson, M. Thomson, W. M. Thong, R. P. Thun, F. Tian, M. J. Tibbetts, V. O. Tikhomirov, Yu. A. Tikhonov, S. Timoshenko, E. Tiouchichine, P. Tipton, S. Tisserant, T. Todorov, S. Todorova-Nova, J. Tojo, S. Tokár, K. Tokushuku, K. Tollefson, E. Tolley, L. Tomlinson, M. Tomoto, L. Tompkins, K. Toms, N. D. Topilin, E. Torrence, H. Torres, E. Torró Pastor, J. Toth, F. Touchard, D. R. Tovey, H. L. Tran, T. Trefzger, L. Tremblet, A. Tricoli, I. M. Trigger, S. Trincaz-Duvoid, M. F. Tripiana, W. Trischuk, B. Trocmé, C. Troncon, M. Trottier-McDonald, M. Trovatelli, P. True, M. Trzebinski, A. Trzupek, C. Tsarouchas, J. C.-L. Tseng, P. V. Tsiareshka, D. Tsionou, G. Tsipolitis, N. Tsirintanis, S. Tsiskaridze, V. Tsiskaridze, E. G. Tskhadadze, I. I. Tsukerman, V. Tsulaia, S. Tsuno, D. Tsybychev, A. Tudorache, V. Tudorache, A. N. Tuna, S. A. Tupputi, S. Turchikhin, D. Turecek, I. Turk Cakir, R. Turra, A. J. Turvey, P. M. Tuts, A. Tykhonov, M. Tylmad, M. Tyndel, I. Ueda, R. Ueno, M. Ughetto, M. Ugland, M. Uhlenbrock, F. Ukegawa, G. Unal, A. Undrus, G. Unel, F. C. Ungaro, Y. Unno, C. Unverdorben, J. Urban, D. Urbaniec, P. Urquijo, G. Usai, A. Usanova, L. Vacavant, V. Vacek, B. Vachon, N. Valencic, S. Valentinetti, A. Valero, L. Valery, S. Valkar, E. Valladolid Gallego, S. Vallecorsa, J. A. Valls Ferrer, W. Van Den Wollenberg, P. C. Van Der Deijl, R. van der Geer, H. van der Graaf, R. Van Der Leeuw, D. van der Ster, N. van Eldik, P. van Gemmeren, J. Van Nieuwkoop, I. van Vulpen, M. C. van Woerden, M. Vanadia, W. Vandelli, R. Vanguri, A. Vaniachine, P. Vankov, F. Vannucci, G. Vardanyan, R. Vari, E. W. Varnes, T. Varol, D. Varouchas, A. Vartapetian, K. E. Varvell, F. Vazeille, T. Vazquez Schroeder, J. Veatch, F. Veloso, T. Velz, S. Veneziano, A. Ventura, D. Ventura, M. Venturi, N. Venturi, A. Venturini, V. Vercesi, M. Verducci, W. Verkerke, J. C. Vermeulen, A. Vest, M. C. Vetterli, O. Viazlo, I. Vichou, T. Vickey, O. E. Vickey Boeriu, G. H. A. Viehhauser, S. Viel, R. Vigne, M. Villa, M. Villaplana Perez, E. Vilucchi, M. G. Vincter, V. B. Vinogradov, J. Virzi, I. Vivarelli, F. Vives Vaque, S. Vlachos, D. Vladoiu, M. Vlasak, A. Vogel, M. Vogel, P. Vokac, G. Volpi, M. Volpi, H. von der Schmitt, H. von Radziewski, E. von Toerne, V. Vorobel, K. Vorobev, M. Vos, R. Voss, J. H. Vossebeld, N. Vranjes, M. Vranjes Milosavljevic, V. Vrba, M. Vreeswijk, T. Vu Anh, R. Vuillermet, I. Vukotic, Z. Vykydal, P. Wagner, W. Wagner, H. Wahlberg, S. Wahrmund, J. Wakabayashi, J. Walder, R. Walker, W. Walkowiak, R. Wall, P. Waller, B. Walsh, C. Wang, C. Wang, F. Wang, H. Wang, H. Wang, J. Wang, J. Wang, K. Wang, R. Wang, S. M. Wang, T. Wang, X. Wang, C. Wanotayaroj, A. Warburton, C. P. Ward, D. R. Wardrope, M. Warsinsky, A. Washbrook, C. Wasicki, P. M. Watkins, A. T. Watson, I. J. Watson, M. F. Watson, G. Watts, S. Watts, B. M. Waugh, S. Webb, M. S. Weber, S. W. Weber, J. S. Webster, A. R. Weidberg, B. Weinert, J. Weingarten, C. Weiser, H. Weits, P. S. Wells, T. Wenaus, D. Wendland, Z. Weng, T. Wengler, S. Wenig, N. Wermes, M. Werner, P. Werner, M. Wessels, J. Wetter, K. Whalen, A. White, M. J. White, R. White, S. White, D. Whiteson, D. Wicke, F. J. Wickens, W. Wiedenmann, M. Wielers, P. Wienemann, C. Wiglesworth, L. A. M. Wiik-Fuchs, P. A. Wijeratne, A. Wildauer, M. A. Wildt, H. G. Wilkens, H. H. Williams, S. Williams, C. Willis, S. Willocq, A. Wilson, J. A. Wilson, I. Wingerter-Seez, F. Winklmeier, B. T. Winter, M. Wittgen, J. Wittkowski, S. J. Wollstadt, M. W. Wolter, H. Wolters, B. K. Wosiek, J. Wotschack, M. J. Woudstra, K. W. Wozniak, M. Wright, M. Wu, S. L. Wu, X. Wu, Y. Wu, E. Wulf, T. R. Wyatt, B. M. Wynne, S. Xella, M. Xiao, D. Xu, L. Xu, B. Yabsley, S. Yacoob, R. Yakabe, M. Yamada, H. Yamaguchi, Y. Yamaguchi, A. Yamamoto, S. Yamamoto, T. Yamamura, T. Yamanaka, K. Yamauchi, Y. Yamazaki, Z. Yan, H. Yang, H. Yang, Y. Yang, S. Yanush, L. Yao, W.-M. Yao, Y. Yasu, E. Yatsenko, K. H. Yau Wong, J. Ye, S. Ye, I. Yeletskikh, A. L. Yen, E. Yildirim, M. Yilmaz, R. Yoosoofmiya, K. Yorita, R. Yoshida, K. Yoshihara, C. Young, C. J. S. Young, S. Youssef, D. R. Yu, J. Yu, J. M. Yu, J. Yu, L. Yuan, A. Yurkewicz, I. Yusuff, B. Zabinski, R. Zaidan, A. M. Zaitsev, A. Zaman, S. Zambito, L. Zanello, D. Zanzi, C. Zeitnitz, M. Zeman, A. Zemla, K. Zengel, O. Zenin, T. Ženiš, D. Zerwas, G. Zevi della Porta, D. Zhang, F. Zhang, H. Zhang, J. Zhang, L. Zhang, R. Zhang, X. Zhang, Z. Zhang, Y. Zhao, Z. Zhao, A. Zhemchugov, J. Zhong, B. Zhou, L. Zhou, L. Zhou, N. Zhou, C. G. Zhu, H. Zhu, J. Zhu, Y. Zhu, X. Zhuang, K. Zhukov, A. Zibell, D. Zieminska, N. I. Zimine, C. Zimmermann, R. Zimmermann, S. Zimmermann, S. Zimmermann, Z. Zinonos, M. Ziolkowski, G. Zobernig, A. Zoccoli, M. zur Nedden, G. Zurzolo, L. Zwalinski

**Affiliations:** 1 Department of Physics, University of Adelaide, Adelaide, Australia; 2 Physics Department, SUNY Albany, Albany, NY USA; 3 Department of Physics, University of Alberta, Edmonton, AB Canada; 4 Department of Physics, Ankara University, Ankara, Turkey; Department of Physics, Gazi University, Ankara, Turkey; Istanbul Aydin University, Istanbul; Division of Physics, TOBB University of Economics and Technology, Ankara, Turkey; 5 LAPP, CNRS/IN2P3 and Université de Savoie, Annecy-le-Vieux, France; 6 High Energy Physics Division, Argonne National Laboratory, Argonne, IL USA; 7 Department of Physics, University of Arizona, Tucson, AZ USA; 8 Department of Physics, The University of Texas at Arlington, Arlington, TX USA; 9 Physics Department, University of Athens, Athens, Greece; 10 Physics Department, National Technical University of Athens, Zografou, Greece; 11 Institute of Physics, Azerbaijan Academy of Sciences, Baku, Azerbaijan; 12 Institut de Física d’Altes Energies and Departament de Física de la Universitat Autònoma de Barcelona, Barcelona, Spain; 13 Institute of Physics, University of Belgrade, Belgrade, Serbia; Vinca Institute of Nuclear Sciences, University of Belgrade, Belgrade, Serbia; 14 Department for Physics and Technology, University of Bergen, Bergen, Norway; 15 Physics Division, Lawrence Berkeley National Laboratory and University of California, Berkeley, CA USA; 16 Department of Physics, Humboldt University, Berlin, Germany; 17 Albert Einstein Center for Fundamental Physics and Laboratory for High Energy Physics, University of Bern, Bern, Switzerland; 18 School of Physics and Astronomy, University of Birmingham, Birmingham, UK; 19 Department of Physics, Bogazici University, Istanbul, Turkey; Department of Physics, Dogus University, Istanbul, Turkey; Department of Physics Engineering, Gaziantep University, Gaziantep, Turkey; 20 INFN Sezione di Bologna, Bologna, Italy; Dipartimento di Fisica e Astronomia, Università di Bologna, Bologna, Italy; 21 Physikalisches Institut, University of Bonn, Bonn, Germany; 22 Department of Physics, Boston University, Boston, MA USA; 23 Department of Physics, Brandeis University, Waltham, MA USA; 24 Universidade Federal do Rio De Janeiro COPPE/EE/IF, Rio de Janeiro, Brazil; Electrical Circuits Department, Federal University of Juiz de Fora (UFJF), Juiz de Fora, Brazil; Federal University of Sao Joao del Rei (UFSJ), Sao Joao del Rei, Brazil; Instituto de Fisica, Universidade de Sao Paulo, São Paulo, Brazil; 25 Physics Department, Brookhaven National Laboratory, Upton, NY USA; 26 National Institute of Physics and Nuclear Engineering, Bucharest, Romania; Physics Department, National Institute for Research and Development of Isotopic and Molecular Technologies, Cluj Napoca, Romania; University Politehnica Bucharest, Bucharest, Romania; West University in Timisoara, Timisoara, Romania; 27 Departamento de Física, Universidad de Buenos Aires, Buenos Aires, Argentina; 28 Cavendish Laboratory, University of Cambridge, Cambridge, UK; 29 Department of Physics, Carleton University, Ottawa, ON Canada; 30 CERN, Geneva, Switzerland; 31 Enrico Fermi Institute, University of Chicago, Chicago, IL USA; 32 Departamento de Física, Pontificia Universidad Católica de Chile, Santiago, Chile; Departamento de Física, Universidad Técnica Federico Santa María, Valparaiso, Chile; 33 Institute of High Energy Physics, Chinese Academy of Sciences, Beijing, China; Department of Modern Physics, University of Science and Technology of China, Hefei, Anhui, China; Department of Physics, Nanjing University, Nanjing, Jiangsu, China; School of Physics, Shandong University, Jinan, Shandong, China; Physics Department, Shanghai Jiao Tong University, Shanghai; Physics Department, Tsinghua University, Beijing, 100084 China; 34 Laboratoire de Physique Corpusculaire, Clermont Université and Université Blaise Pascal and CNRS/IN2P3, Clermont-Ferrand, France; 35 Nevis Laboratory, Columbia University, Irvington, NY USA; 36 Niels Bohr Institute, University of Copenhagen, Kobenhavn, Denmark; 37 INFN Gruppo Collegato di Cosenza, Laboratori Nazionali di Frascati, Frascati, Italy; Dipartimento di Fisica, Università della Calabria, Rende, Italy; 38 Faculty of Physics and Applied Computer Science, AGH University of Science and Technology, Kraków, Poland; Marian Smoluchowski Institute of Physics, Jagiellonian University, Kraków, Poland; 39 The Henryk Niewodniczanski Institute of Nuclear Physics, Polish Academy of Sciences, Kraków, Poland; 40 Physics Department, Southern Methodist University, Dallas, TX USA; 41 Physics Department, University of Texas at Dallas, Richardson, TX USA; 42 DESY, Hamburg and Zeuthen, Germany; 43 Institut für Experimentelle Physik IV, Technische Universität Dortmund, Dortmund, Germany; 44 Institut für Kern- und Teilchenphysik, Technische Universität Dresden, Dresden, Germany; 45 Department of Physics, Duke University, Durham, NC USA; 46 SUPA-School of Physics and Astronomy, University of Edinburgh, Edinburgh, UK; 47 INFN Laboratori Nazionali di Frascati, Frascati, Italy; 48 Fakultät für Mathematik und Physik, Albert-Ludwigs-Universität, Freiburg, Germany; 49 Section de Physique, Université de Genève, Geneva, Switzerland; 50 INFN Sezione di Genova; Dipartimento di Fisica, Università di Genova, Genova, Italy; 51 E. Andronikashvili Institute of Physics, Iv. Javakhishvili Tbilisi State University, Tbilisi, Georgia; High Energy Physics Institute, Tbilisi State University, Tbilisi, Georgia; 52 II Physikalisches Institut, Justus-Liebig-Universität Giessen, Giessen, Germany; 53 SUPA-School of Physics and Astronomy, University of Glasgow, Glasgow, UK; 54 II Physikalisches Institut, Georg-August-Universität, Göttingen, Germany; 55 Laboratoire de Physique Subatomique et de Cosmologie, Université Grenoble-Alpes, CNRS/IN2P3, Grenoble, France; 56 Department of Physics, Hampton University, Hampton, VA USA; 57 Laboratory for Particle Physics and Cosmology, Harvard University, Cambridge, MA USA; 58 Kirchhoff-Institut für Physik, Ruprecht-Karls-Universität Heidelberg, Heidelberg, Germany; Physikalisches Institut, Ruprecht-Karls-Universität Heidelberg, Heidelberg, Germany; ZITI Institut für technische Informatik, Ruprecht-Karls-Universität Heidelberg, Mannheim, Germany; 59 Faculty of Applied Information Science, Hiroshima Institute of Technology, Hiroshima, Japan; 60 Department of Physics, The Chinese University of Hong Kong, Shatin, N.T., Hong Kong; Department of Physics, The University of Hong Kong, Hong Kong; Department of Physics, The Hong Kong University of Science and Technology, Clear Water Bay, Kowloon, Hong Kong China; 61 Department of Physics, Indiana University, Bloomington, IN USA; 62 Institut für Astro- und Teilchenphysik, Leopold-Franzens-Universität, Innsbruck, Austria; 63 University of Iowa, Iowa City, IA USA; 64 Department of Physics and Astronomy, Iowa State University, Ames, IA USA; 65 Joint Institute for Nuclear Research, JINR Dubna, Dubna, Russia; 66 KEK, High Energy Accelerator Research Organization, Tsukuba, Japan; 67 Graduate School of Science, Kobe University, Kobe, Japan; 68 Faculty of Science, Kyoto University, Kyoto, Japan; 69 Kyoto University of Education, Kyoto, Japan; 70 Department of Physics, Kyushu University, Fukuoka, Japan; 71 Instituto de Física La Plata, Universidad Nacional de La Plata and CONICET, La Plata, Argentina; 72 Physics Department, Lancaster University, Lancaster, UK; 73 INFN Sezione di Lecce, Lecce, Italy; Dipartimento di Matematica e Fisica, Università del Salento, Lecce, Italy; 74 Oliver Lodge Laboratory, University of Liverpool, Liverpool, UK; 75 Department of Physics, Jožef Stefan Institute and University of Ljubljana, Ljubljana, Slovenia; 76 School of Physics and Astronomy, Queen Mary University of London, London, UK; 77 Department of Physics, Royal Holloway University of London, Surrey, UK; 78 Department of Physics and Astronomy, University College London, London, UK; 79 Louisiana Tech University, Ruston, LA USA; 80 Laboratoire de Physique Nucléaire et de Hautes Energies, UPMC and Université Paris-Diderot and CNRS/IN2P3, Paris, France; 81 Fysiska institutionen, Lunds universitet, Lund, Sweden; 82 Departamento de Fisica Teorica C-15, Universidad Autonoma de Madrid, Madrid, Spain; 83 Institut für Physik, Universität Mainz, Mainz, Germany; 84 School of Physics and Astronomy, University of Manchester, Manchester, UK; 85 CPPM, Aix-Marseille Université and CNRS/IN2P3, Marseille, France; 86 Department of Physics, University of Massachusetts, Amherst, MA USA; 87 Department of Physics, McGill University, Montreal, QC Canada; 88 School of Physics, University of Melbourne, Parkville, VIC Australia; 89 Department of Physics, The University of Michigan, Ann Arbor, MI USA; 90 Department of Physics and Astronomy, Michigan State University, East Lansing, MI USA; 91 INFN Sezione di Milano, Milan, Italy; Dipartimento di Fisica, Università di Milano, Milan, Italy; 92 B.I. Stepanov Institute of Physics, National Academy of Sciences of Belarus, Minsk, Republic of Belarus; 93 National Scientific and Educational Centre for Particle and High Energy Physics, Minsk, Republic of Belarus; 94 Department of Physics, Massachusetts Institute of Technology, Cambridge, MA USA; 95 Group of Particle Physics, University of Montreal, Montreal, QC Canada; 96 P.N. Lebedev Institute of Physics, Academy of Sciences, Moscow, Russia; 97 Institute for Theoretical and Experimental Physics (ITEP), Moscow, Russia; 98 National Research Nuclear University MEPhI, Moscow, Russia; 99 D.V. Skobeltsyn Institute of Nuclear Physics, M.V. Lomonosov Moscow State University, Moscow, Russia; 100 Fakultät für Physik, Ludwig-Maximilians-Universität München, Munich, Germany; 101 Max-Planck-Institut für Physik (Werner-Heisenberg-Institut), Munich, Germany; 102 Nagasaki Institute of Applied Science, Nagasaki, Japan; 103 Graduate School of Science and Kobayashi-Maskawa Institute, Nagoya University, Nagoya, Japan; 104 INFN Sezione di Napoli, Napoli, Italy; Dipartimento di Fisica, Università di Napoli, Naples, Italy; 105 Department of Physics and Astronomy, University of New Mexico, Albuquerque, NM USA; 106 Institute for Mathematics, Astrophysics and Particle Physics, Radboud University Nijmegen/Nikhef, Nijmegen, The Netherlands; 107 Nikhef National Institute for Subatomic Physics and University of Amsterdam, Amsterdam, The Netherlands; 108 Department of Physics, Northern Illinois University, DeKalb, IL USA; 109 Budker Institute of Nuclear Physics, SB RAS, Novosibirsk, Russia; 110 Department of Physics, New York University, New York, NY USA; 111 Ohio State University, Columbus, OH USA; 112 Faculty of Science, Okayama University, Okayama, Japan; 113 Homer L. Dodge Department of Physics and Astronomy, University of Oklahoma, Norman, OK USA; 114 Department of Physics, Oklahoma State University, Stillwater, OK USA; 115 Palacký University, RCPTM, Olomouc, Czech Republic; 116 Center for High Energy Physics, University of Oregon, Eugene, OR USA; 117 LAL, Université Paris-Sud and CNRS/IN2P3, Orsay, France; 118 Graduate School of Science, Osaka University, Osaka, Japan; 119 Department of Physics, University of Oslo, Oslo, Norway; 120 Department of Physics, Oxford University, Oxford, UK; 121 INFN Sezione di Pavia, Pavia, Italy; Dipartimento di Fisica, Università di Pavia, Pavia, Italy; 122 Department of Physics, University of Pennsylvania, Philadelphia, PA USA; 123 Petersburg Nuclear Physics Institute, Gatchina, Russia; 124 INFN Sezione di Pisa, Pisa, Italy; Dipartimento di Fisica E. Fermi, Università di Pisa, Pisa, Italy; 125 Department of Physics and Astronomy, University of Pittsburgh, Pittsburgh, PA USA; 126 Laboratorio de Instrumentacao e Fisica Experimental de Particulas-LIP, Lisbon, Portugal; Faculdade de Ciências, Universidade de Lisboa, Lisbon, Portugal; Department of Physics, University of Coimbra, Coimbra, Portugal; Centro de Física Nuclear da Universidade de Lisboa, Lisbon, Portugal; Departamento de Fisica, Universidade do Minho, Braga, Portugal; Departamento de Fisica Teorica y del Cosmos and CAFPE, Universidad de Granada, Granada, Spain; Dep Fisica and CEFITEC of Faculdade de Ciencias e Tecnologia, Universidade Nova de Lisboa, Caparica, Portugal; 127 Institute of Physics, Academy of Sciences of the Czech Republic, Praha, Czech Republic; 128 Czech Technical University in Prague, Praha, Czech Republic; 129 Faculty of Mathematics and Physics, Charles University in Prague, Praha, Czech Republic; 130 State Research Center Institute for High Energy Physics, Protvino, Russia; 131 Particle Physics Department, Rutherford Appleton Laboratory, Didcot, UK; 132 Ritsumeikan University, Kusatsu, Shiga Japan; 133 INFN Sezione di Roma, Rome, Italy; Dipartimento di Fisica, Sapienza Università di Roma, Rome, Italy; 134 INFN Sezione di Roma Tor Vergata, Rome, Italy; Dipartimento di Fisica, Università di Roma Tor Vergata, Rome, Italy; 135 INFN Sezione di Roma Tre, Rome, Italy; Dipartimento di Matematica e Fisica, Università Roma Tre, Rome, Italy; 136 Faculté des Sciences Ain Chock, Réseau Universitaire de Physique des Hautes Energies-Université Hassan II, Casablanca, Morocco; Centre National de l’Energie des Sciences Techniques Nucleaires, Rabat, Morocco; Faculté des Sciences Semlalia, Université Cadi Ayyad, LPHEA-Marrakech, Marrakech, Morocco; Faculté des Sciences, Université Mohamed Premier and LPTPM, Oujda, Morocco; Faculté des Sciences, Université Mohammed V-Agdal, Rabat, Morocco; 137 DSM/IRFU (Institut de Recherches sur les Lois Fondamentales de l’Univers), CEA Saclay (Commissariat à l’Energie Atomique et aux Energies Alternatives), Gif-sur-Yvette, France; 138 Santa Cruz Institute for Particle Physics, University of California Santa Cruz, Santa Cruz, CA USA; 139 Department of Physics, University of Washington, Seattle, WA USA; 140 Department of Physics and Astronomy, University of Sheffield, Sheffield, UK; 141 Department of Physics, Shinshu University, Nagano, Japan; 142 Fachbereich Physik, Universität Siegen, Siegen, Germany; 143 Department of Physics, Simon Fraser University, Burnaby, BC Canada; 144 SLAC National Accelerator Laboratory, Stanford, CA USA; 145 Faculty of Mathematics, Physics and Informatics, Comenius University, Bratislava, Slovak Republic; Department of Subnuclear Physics, Institute of Experimental Physics of the Slovak Academy of Sciences, Kosice, Slovak Republic; 146 Department of Physics, University of Cape Town, Cape Town, South Africa; Department of Physics, University of Johannesburg, Johannesburg, South Africa; School of Physics, University of the Witwatersrand, Johannesburg, South Africa; 147 Department of Physics, Stockholm University, Stockholm, Sweden; The Oskar Klein Centre, Stockholm, Sweden; 148 Physics Department, Royal Institute of Technology, Stockholm, Sweden; 149 Departments of Physics and Astronomy and Chemistry, Stony Brook University, Stony Brook, NY USA; 150 Department of Physics and Astronomy, University of Sussex, Brighton, UK; 151 School of Physics, University of Sydney, Sydney, Australia; 152 Institute of Physics, Academia Sinica, Taipei, Taiwan; 153 Department of Physics, Technion: Israel Institute of Technology, Haifa, Israel; 154 Raymond and Beverly Sackler School of Physics and Astronomy, Tel Aviv University, Tel Aviv, Israel; 155 Department of Physics, Aristotle University of Thessaloniki, Thessaloniki, Greece; 156 International Center for Elementary Particle Physics and Department of Physics, The University of Tokyo, Tokyo, Japan; 157 Graduate School of Science and Technology, Tokyo Metropolitan University, Tokyo, Japan; 158 Department of Physics, Tokyo Institute of Technology, Tokyo, Japan; 159 Department of Physics, University of Toronto, Toronto, ON Canada; 160 TRIUMF, Vancouver, BC, Canada; Department of Physics and Astronomy, York University, Toronto, ON Canada; 161 Faculty of Pure and Applied Sciences, University of Tsukuba, Tsukuba, Japan; 162 Department of Physics and Astronomy, Tufts University, Medford, MA USA; 163 Centro de Investigaciones, Universidad Antonio Narino, Bogota, Colombia; 164 Department of Physics and Astronomy, University of California Irvine, Irvine, CA USA; 165 INFN Gruppo Collegato di Udine, Sezione di Trieste, Udine, Italy; ICTP, Trieste, Italy; Dipartimento di Chimica, Fisica e Ambiente, Università di Udine, Udine, Italy; 166 Department of Physics, University of Illinois, Urbana, IL USA; 167 Department of Physics and Astronomy, University of Uppsala, Uppsala, Sweden; 168 Instituto de Física Corpuscular (IFIC) and Departamento de Física Atómica, Molecular y Nuclear and Departamento de Ingeniería Electrónica and Instituto de Microelectrónica de Barcelona (IMB-CNM), University of Valencia and CSIC, Valencia, Spain; 169 Department of Physics, University of British Columbia, Vancouver, BC Canada; 170 Department of Physics and Astronomy, University of Victoria, Victoria, BC Canada; 171 Department of Physics, University of Warwick, Coventry, UK; 172 Waseda University, Tokyo, Japan; 173 Department of Particle Physics, The Weizmann Institute of Science, Rehovot, Israel; 174 Department of Physics, University of Wisconsin, Madison, WI USA; 175 Fakultät für Physik und Astronomie, Julius-Maximilians-Universität, Würzburg, Germany; 176 Fachbereich C Physik, Bergische Universität Wuppertal, Wuppertal, Germany; 177 Department of Physics, Yale University, New Haven, CT USA; 178 Yerevan Physics Institute, Yerevan, Armenia; 179 Centre de Calcul de l’Institut National de Physique Nucléaire et de Physique des Particules (IN2P3), Villeurbanne, France; 180 CERN, 1211 Geneva 23, Switzerland

## Abstract

A search for the production of single-top-quarks in association with missing energy is performed in proton–proton collisions at a centre-of-mass energy of $\sqrt{s}=\mathrm {8~TeV}$ with the ATLAS experiment at the large hadron collider using data collected in 2012, corresponding to an integrated luminosity of $20.3$ fb$^{-1}$. In this search, the $W$ boson from the top quark is required to decay into an electron or a muon and a neutrino. No deviation from the standard model prediction is observed, and upper limits are set on the production cross-section for resonant and non-resonant production of an invisible exotic state in association with a right-handed top quark. In the case of resonant production, for a spin-$0$ resonance with a mass of $500$ GeV, an effective coupling strength above $0.15$ is excluded at 95$\,\%$ confidence level for the top quark and an invisible spin-$1/2$ state with mass between $0$ and $100$ GeV. In the case of non-resonant production, an effective coupling strength above $0.2$ is excluded at 95$\,\%$ confidence level for the top quark and an invisible spin-$1$ state with mass between $0$ and $657$ GeV.

## Introduction

Many theories beyond the standard model (BSM) predict enhanced production of events with large missing energy in association with a single reconstructed object. Such events have been searched for at the large hadron collider (LHC), when the single object is either a photon [1, 2], a jet [3, 4], or a $W$ or $Z$ boson [5, 6].

**Fig. 1:**
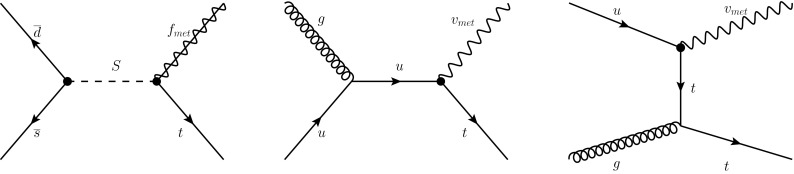
Example of Feynman diagrams of leading-order processes leading to monotop events: (*left*) production of a coloured scalar resonance $S$ decaying into a top quark and a spin-$1/2$ fermion $f_{\mathrm {met}}$ in the resonant model, and (*middle*) $s$- and (*right*) $t$-channel non-resonant production of a top quark in association with a spin-1 boson $v_{\mathrm {met}}$ in the non-resonant model

This paper presents a search for singly produced top quarks in association with significant missing energy, corresponding to the associated production of one or several undetected neutral particles, and without any other reconstructed object. These neutral particles can be either stable and/or weakly interacting with ordinary matter – providing an interesting interpretation in terms of dark-matter candidates – or long-lived and decaying outside of the detector. The observation of such final states, commonly referred to as monotop events, would be evidence for new phenomena. Moreover, processes involving top quarks are sensitive to BSM physics, due to the large mass of this standard model (SM) particle which is close to the electroweak symmetry-breaking scale.

No such process is possible in the SM at tree level: the direct production of a top quark and a $Z$ boson decaying into a pair of neutrinos, without any additional quark, is suppressed by the Glashow–Iliopoulos–Maiani mechanism [7].

This search is performed with the ATLAS detector [8] in $pp$ collisions at $\mathrm {\sqrt{s}=8~TeV}$ with the data collected in 2012 at the LHC and corresponding to an integrated luminosity of $\mathrm {20.3~fb^{-1}}$. The ATLAS detector covers the pseudorapidity range $|\eta | <$ 4.9 and the full azimuthal angle $\phi$.^1^ It consists of an inner tracking detector covering the pseudorapidity range $|\eta | <$ 2.5 surrounded by a superconducting solenoid, electromagnetic and hadronic calorimeters, and an external muon spectrometer with large superconducting toroidal magnets.

The search is based on the analysis of top-quark events where the $W$ boson from the top quark decays into a lepton and a neutrino. Previous results of a search for monotop production, exploiting the case of fully hadronic top-quark decays, have been published by the CDF Collaboration using $p\overline{p}$ collision data at $\mathrm {\sqrt{s}=1.96~TeV}$, corresponding to an integrated luminosity of $\mathrm {7.7~fb^{-1}}$ [9], and more recently by the CMS Collaboration using $pp$ collision data at $\mathrm {\sqrt{s}=8~TeV}$, corresponding to an integrated luminosity of $\mathrm {19.7~fb^{-1}}$ [10].

## Signal models

Many theoretical models predicting the production of monotop events in hadron colliders have been proposed. In a first class of theories, a charged resonance is produced by down-type antiquark fusion and decays into a top quark and a neutral particle, as in $SU(5)$ models [11], in R-parity-violating SUSY [12] or in hylogenesis models [13, 14]. In a second class of theories, the monotop final state is produced through a non-resonant process, as in R-parity-conserving SUSY [15], or in models where an interaction of a gluon with an up-type quark allows production of a set of invisible particles via a $u$–$t$ or $c$–$t$ coupling [16–19].

Because of the variety of these theories, effective models [20, 21] are used for the search reported in this paper. Furthermore, as minimal extensions of the SM, the effective models tested in this search are required to respect the electroweak gauge structure [22]. The possibilities for monotop production in $pp$ collisions considered are thus:

- Resonant production of a +2/3 charged spin-0 boson, $S$, decaying into a right-handed top quark and a neutral, colour singlet, spin-1/2 fermion, $f_{\mathrm {met}}$;
- Non-resonant production of a neutral, colour singlet, spin-1 boson, $v_{\mathrm {met}}$, in association with a right-handed top quark.

The Feynman diagrams for monotop production in the resonant and non-resonant models are shown in Fig. 1. Each of these effective models corresponds to one of the two classes of BSM theories detailed above.

A detailed study of the phenomenology of the resonant model is available in Ref. [23]. The interaction Lagrangians of the resonant and non-resonants models are given in Eqs. (1) and (2), respectively.

1 $$\begin{aligned}&\mathcal{L}_{\mathrm {res}} = \epsilon ^{\alpha \beta \gamma } \ \varphi _{\alpha } \ \overline{d}^{i,\mathrm {c}}_{\mathrm {\beta ,R}} (a_{\mathrm {res}}^q)_{ij} \ d^j_{\gamma ,\mathrm {R}} + \varphi \ \bar{u}^{k}_{\mathrm {R}} \ (a_{\mathrm {res}}^{1/2})_{k} \chi + h.c. \end{aligned}$$

2 $$\begin{aligned}&\mathcal{L}_{\mathrm {non\text{- }res}} = (a_{\mathrm {non\text{- }res}})_{ij} V_{\mu } \overline{u}_{\mathrm {R}}^{i} \gamma ^{\mu } u_{\mathrm {R}}^{j} +h.c. \end{aligned}$$

The fields $\varphi$, $\chi$, and $V_{\mu }$ correspond to the $S$, $f_{\mathrm {met}}$, and $v_{\mathrm {met}}$ exotic particles, respectively, the field $\overline{u}$ ($\overline{d}$) represents an up-type (down-type) quark, $(a_{\mathrm {res}}^q)_{ij}$, $(a_{\mathrm {res}}^{1/2})_{k}$, and $(a_{\mathrm {non\text{- }res}})_{ij}$ are the coupling matrices in the quark-flavour space, the indices $i$, $j$, $k$, represent the quark-generation number, and $\epsilon ^{\alpha \beta \gamma }$ is the fully antisymmetric tensor, the indices $\alpha$, $\beta$, and $\gamma$ being the colour indices. The superscript $^{\mathrm {c}}$ denotes the charge conjugation. The number of free parameters is reduced by assuming $(a_{\mathrm {res}}^q)_{\mathrm {12}}=(a_{\mathrm {res}}^q)_{\mathrm {21}}=(a_{\mathrm {res}}^{1/2})_{\mathrm {3}}\equiv a_{\mathrm {res}}$ for the resonant model and $(a_{\mathrm {non\text{- }res}})_{\mathrm {13}}=(a_{\mathrm {non\text{- }res}})_{\mathrm {31}}\equiv a_{\mathrm {non\text{- }res}}$ for the non-resonant model, all other elements of these coupling matrices being equal to 0. For each model, the coupling parameter $a_{\mathrm {res}}$ or $a_{\mathrm {non\text{- }res}}$ and the masses of the exotic particles are independent.

The choice of model parameters – the effective couplings and the masses of the particles – is driven by phenomenological considerations: the particles $f_{\mathrm {met}}$ and $v_{\mathrm {met}}$ in the resonant and non-resonant models, respectively, are required to have missing transverse momentum as an experimental signature. For the resonant model, in which the $f_{\mathrm {met}}$ fermion can decay into a five-body final state, Ref. [23] suggests that for $m(S)=500$ GeV and an effective coupling of $a_{\mathrm {res}}=0.2$, the decay length of $f_{\mathrm {met}}$ is large enough to be considered as invisible for the detector, as long as $m(f_{\mathrm {met}})$ is below 100 GeV. For the non-resonant model, in which the $v_{\mathrm {met}}$ boson can decay into a two-body final state either through a tree-level or a loop-induced interaction, Ref. [22] assumes that the $v_{\mathrm {met}}$ boson decays into a set of invisible particles which can be dark-matter candidates. This assumption follows the spirit of several BSM models [16–18]. Hence, the $v_{\mathrm {met}}$ particle in the non-resonant model can be considered to be an invisible spin-1 state with mass $m(v_{\mathrm {met}})$. Studies of possible direct and indirect constraints on monotop model parameters using experimental signatures other than monotop processes are discussed in Refs. [22, 23].

## Data and Monte Carlo samples

The data used for this analysis are selected from the recorded data streams using single-electron and single-muon triggers [24]. Stringent detector and data quality criteria are applied offline, resulting in a data sample corresponding to an integrated luminosity of $20.3 \pm 0.6 ~\mathrm{fb}^{-1}$ [25].

The signal samples are generated at leading order (LO) in QCD with the matrix-element generator MadGraph5 v1.5.11 [26] using FeynRules [27–29] and interfaced with Pythia v8.175 [30, 31] for parton showering and hadronisation. The parton distribution function (PDF) set MSTW2008LO [32, 33] is used. Resonant signal samples are generated with the mass of the invisible state $f_{\mathrm {met}}$ varying from 0 to 100 GeV, the mass of the $S$ resonance being fixed at 500 GeV – following the suggestion in Ref. [23] – and non-resonant signal samples are generated with the mass of the invisible state $v_{\mathrm {met}}$ varying from 0 to 1,000 GeV. The couplings $a_{\mathrm {res}}$ and $a_{\mathrm {non\text{- }res}}$ are set at a fixed value of $0.2$. In addition, two samples are produced for the resonant model for $m(f_{\mathrm {met}})=100$ GeV, with coupling strengths fixed at $a_{\mathrm {res}}=0.5$ and $a_{\mathrm {res}}=1.0$, in order to check the effect of the resonance width on the signal event kinematics. The total width of the resonance varies quadratically with the coupling strength, corresponding to a width of 3.5, 21.6, and 86.5 GeV at $a_{\mathrm {res}}=0.2$, $a_{\mathrm {res}}=0.5$, and $a_{\mathrm {res}}=1.0$, respectively.

Top-quark pair ($t\bar{t}$) and single-top $s$-channel and $Wt$ events are simulated using the next-to-leading order (NLO) generator Powheg-Box v1_r2129, v1_r1556, and v1_r2092, respectively [34, 35], with CT10 PDF [36, 37]. The $t$-channel single-top events are generated using the LO AcerMC generator v3.8 [38, 39], with the CTEQ6L1 PDF [40]. The parton showering, the hadronisation, and the underlying event are modelled using Pythia v6.426 [30].

**Fig. 2:**
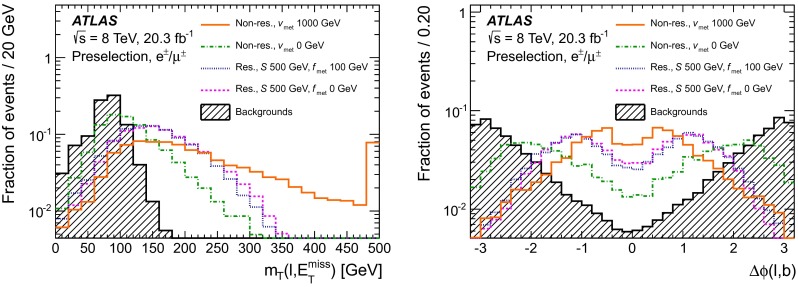
Distributions normalised to unity of (*left*) $m_{\mathrm {T}}(\ell , E_{\mathrm {T}}^{\mathrm {miss}})$ and of (*right*) $\Delta \phi (\ell ,b)$ for events satisfying the pre-selection defined in the text. The expected distributions for the resonant model with $m(S)=500$ GeV are shown for the $m(f_\mathrm{met})=0$ GeV and $m(f_{\mathrm {met}})=100$ GeV hypotheses, as well as for the non-resonant model for the $m(v_{\mathrm {met}})=0$ GeV and $m(v_{\mathrm {met}})=1{,}000$ GeV hypotheses. All distributions are compared to the expected distribution for the backgrounds. For the $m_{\mathrm {T}}(\ell , E_{\mathrm {T}}^{\mathrm {miss}})$ distributions, the last bin includes overflows

The $t\bar{t}$ cross-section for $pp$ collisions at a centre-of-mass energy of $\sqrt{s} = 8 {\mathrm {\ TeV}}$ is $\sigma _{t\bar{t}}= 253^{+13}_{-15}$ pb for a top-quark mass of $172.5$ GeV. It has been calculated at next-to-next-to-leading order (NNLO) in QCD including resummation of next-to-next-to-leading logarithmic (NNLL) soft gluon terms [41–46] with the program Top++ v2.0 [47]. The PDF and $\alpha _S$ uncertainties were calculated using the PDF4LHC prescription [48] with the MSTW2008 68 % CL NNLO [32, 33], CT10 NNLO [36, 37] and NNPDF2.3 5f FFN [49] PDF sets, added in quadrature to the scale uncertainty. The single-top cross-sections are obtained from approximate NNLO calculations: 87.8$^{+3.4}_{-1.9}$ pb ($t$-channel), 22.4$\pm$1.5 pb ($Wt$ process) and 5.6$\pm$0.2 pb ($s$-channel) [50–52].

The Alpgen LO generator v2.14 [53] is used with Pythia v6.426 to generate events with a $W$ boson produced in association with light or heavy quarks ($W$+light-quarks, $W$+$bb$, $W$+$cc$, $W$+$c$) and $Z$+jets events. The Alpgen matrix elements include diagrams with up to five additional partons. To remove overlaps between the $n$ and $n+1$ parton samples the MLM matching scheme [54] is used. Double counting between the $W$+$n$ parton samples and samples with associated heavy-quark pair production is removed utilising an overlap removal based on a $\Delta R=\sqrt{(\Delta \eta )^2+(\Delta \phi )^2}$ matching criterion. Diboson samples ($WW$, $ZZ$, and $WZ$) where at least one of the bosons decays leptonically are modelled by Herwig v6.52 [55]. The single-boson and diboson simulation samples are normalised to the production cross-sections calculated at NNLO [56, 57] and NLO [58], respectively.

After event generation, all signal and background samples are passed through the full simulation of the ATLAS detector [59] based on GEANT4 [60] and reconstructed using the same procedure as for collision data. All Monte Carlo (MC) samples are simulated with pile-up^2^ and re-weighted to have the same distribution of the mean number of interactions per bunch-crossing as in the data sample (20.7 on average).

## Selection and background estimation

The experimental signature of the monotop events is one isolated charged lepton (electron or muon) from the $W$ decay, large missing transverse momentum, and one jet identified as likely to have originated from a $b$-quark ($b$-tagged).

Electrons are identified as energy clusters in the electromagnetic calorimeter matched to reconstructed tracks in the inner detector [61–63]. Electron candidates are required to be isolated from other objects in the event and from hadronic activity to reduce the contamination by mis-reconstructed hadrons, electrons from heavy-flavour decays, and photon conversions. Muons are reconstructed using information from the muon spectrometer and the inner detector [64]. An isolation criterion [65] is applied to reduce the contribution of muons from heavy-flavour decays. The reconstructed charged lepton is required to have a transverse momentum $p_{\text {T}}$ $>$ 30 GeV to ensure a constant trigger efficiency and to have $|\eta |<$ 2.5 for muons and $|\eta |<$ 2.47 for electrons (for the latter, the electromagnetic calorimeter barrel–endcap transition region 1.37 $<|\eta |<$ 1.52 is excluded).

Jets are reconstructed using the anti-$k_t$ algorithm [66] with a radius parameter $R=0.4$ and calibrated to the hadronic energy scale [67]. Jets are required to have $p_{\text {T}}$ $> 25$ GeV and $\left| \eta \right| < 2.5$. To suppress jets from in-time pileup, at least 50 % of the scalar $p_{\mathrm {T}}$ sum of the tracks associated with a jet is required to be from tracks associated with the primary vertex. This jet vertex fraction requirement is applied only for jets with $p_{\text {T}}$ $<50$ GeV and $|\eta |<2.4$.

Exactly one jet is selected, and is required to be $b$-tagged. The $b$-tagging techniques are based on properties specific to $b$-hadrons, such as long lifetime and large mass [68]. This analysis uses a neural-network-based $b$-tagger which combines several $b$-tagging algorithms. The chosen working point corresponds to a $b$-tagging efficiency of 57 % and a light-quark selection efficiency of 0.2 %, as obtained in simulated $t\bar{t}$ events.

The missing transverse momentum (with magnitude $E_{\mathrm {T}}^{\mathrm {miss}}$) is the negative vector sum of the transverse momentum associated with topological clusters of energy deposits in calorimeter cells and is further refined with object-level corrections from identified electrons, muons, and jets [69, 70]. This analysis requires events to have $E_{\mathrm {T}}^{\mathrm {miss}}$ larger than 35 GeV to reduce the multijet background.

The main background to this final-state selection are $t\bar{t}$ pairs where both top quarks decay semi-leptonically, $t\rightarrow \ell \nu b$, with large $E_{\mathrm {T}}^{\mathrm {miss}}$ due to one lepton and one jet not being reconstructed, and $W$+jets production, particularly with jets from heavy-flavour quarks. The background from multijet production due to misidentification as leptons is reduced by imposing a requirement on the sum of the $E_{\mathrm {T}}^{\mathrm {miss}}$ and the transverse mass^3^ of the lepton–$E_{\mathrm {T}}^{\mathrm {miss}}$ system: $m_{\mathrm {T}}(\ell , E_{\mathrm {T}}^{\mathrm {miss}})+ E_{\mathrm {T}}^{\mathrm {miss}}>~60~\mathrm {{\mathrm {\ GeV}}}$. The distributions of kinematic variables and their normalisation for the multijet background are estimated with a data-driven matrix method [71]. All remaining background processes ($t\bar{t}$, single-top, $W$+jets, $Z$+jets and diboson production) are modelled using simulated samples and are scaled to the theory predictions described in Sect. 3. Possible contributions from $t\bar{t}Z$ and $tZ$ processes [72] in the $Z\rightarrow \nu \nu$ decay mode are found to be negligible.

A counting experiment approach is followed. The monotop signal is prominent in regions of the phase space characterised by high $m_{\mathrm {T}}(\ell , E_{\mathrm {T}}^{\mathrm {miss}})$ values, as suggested by Refs. [18, 21]. Hence, in addition to the pre-selection described previously, a criterion requiring $m_{\mathrm {T}}(\ell , E_{\mathrm {T}}^{\mathrm {miss}})>150~\mathrm {{\mathrm {\ GeV}}}$ is used to define the signal region. In order to improve the sensitivity of the search, an optimisation of the event selection is performed with simulated data, using well-modelled variables. The lepton and the $b$-tagged jet are closer to each other when originating from the decay of a top quark than in the case of $W$+jets and multijet background events. Hence, a criterion imposing the rejection of events with large values of the difference in azimuth between the lepton and the $b$-tagged jet $|\Delta \phi (\ell ,b)|$ is tested, together with increased $m_{\mathrm {T}}(\ell , E_{\mathrm {T}}^{\mathrm {miss}})$ threshold values. Figure 2 shows the distributions of these two variables for the expected background contribution, and for two mass hypotheses considered for each signal model. For each set of cuts on $m_{\mathrm {T}}(\ell , E_{\mathrm {T}}^{\mathrm {miss}})$ and $|\Delta \phi (\ell ,b)|$, the sensitivity is estimated by calculating the expected limit on the production cross-section with the procedure described in Sect. 6 including the systematic uncertainties detailed in Sect. 5. The optimisation was performed using one mass hypothesis $m(f_{\mathrm {met}})=\mathrm {100~GeV}$ for the resonant model, for which the kinematic distributions have only small variations in the studied mass range. For the non-resonant model, characterised by larger variations of the kinematic distributions with $v_{\mathrm {met}}$, four signal mass hypotheses were studied: $m(v_{\mathrm {met}})=0$, 100, 300, and 600 GeV. The resulting best-performing selections, for the tested mass hypotheses, are:

- SRI (resonant model optimisation): $m_{\mathrm {T}}(\ell , E_{\mathrm {T}}^{\mathrm {miss}})>\mathrm {210~GeV}$ and $|\Delta \phi (\ell ,b)|<1.2$
- SRII (non-resonant model optimisation): $m_{\mathrm {T}}(\ell , E_{\mathrm {T}}^{\mathrm {miss}})>\mathrm {250~GeV}$ and $|\Delta \phi (\ell ,b)|<1.4$

**Fig. 3:**
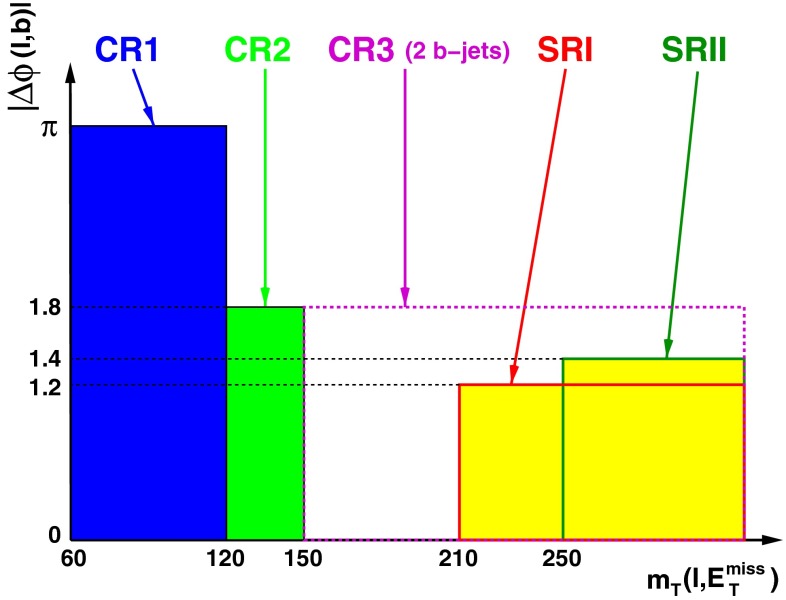
Sketch depicting the control and signal regions in the ($m_{\mathrm {T}}(\ell , E_{\mathrm {T}}^{\mathrm {miss}})$, $|\Delta \phi (\ell ,b)|$)-space. The CR1 (CR2) control region is defined as $60\mathrm {~GeV}<m_{\mathrm {T}}(\ell , E_{\mathrm {T}}^{\mathrm {miss}})<120\mathrm {~GeV}$ ($120\mathrm {~GeV}<m_{\mathrm {T}}(\ell , E_{\mathrm {T}}^{\mathrm {miss}})<150\mathrm {~GeV}$ and $|\Delta \phi (\ell ,b)|<1.8$). The CR3 control region corresponds to $m_{\mathrm {T}}(\ell , E_{\mathrm {T}}^{\mathrm {miss}})>150\mathrm {~GeV}$ and $|\Delta \phi (\ell ,b)|<1.8$, but with a second $b$-tagged jet. The SRI (SRII) signal selection optimised for the resonant (non-resonant) signal model, is defined as $m_{\mathrm {T}}(\ell , E_{\mathrm {T}}^{\mathrm {miss}})>\mathrm {210~GeV}$ and $|\Delta \phi (\ell ,b)|<1.2$ ($m_{\mathrm {T}}(\ell , E_{\mathrm {T}}^{\mathrm {miss}})>\mathrm {250~GeV}$ and $|\Delta \phi (\ell ,b)|<1.4$)

**Fig. 4:**
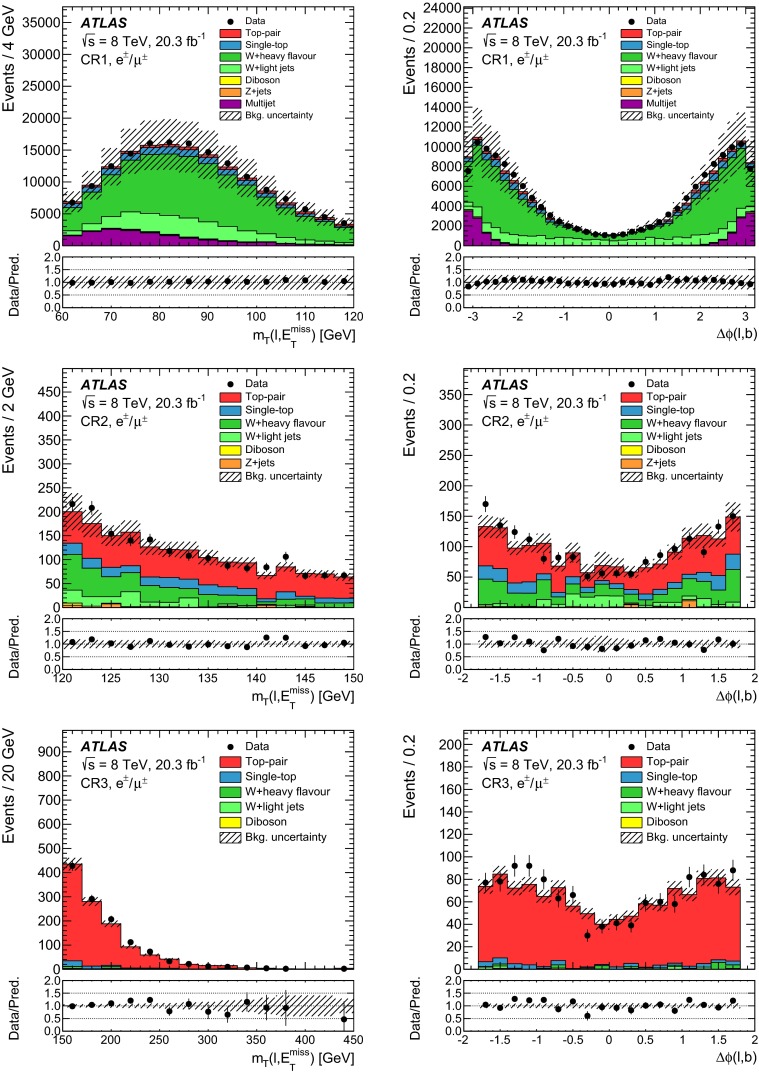
Distributions of (*left*) $m_{\mathrm {T}}(\ell , E_{\mathrm {T}}^{\mathrm {miss}})$ and of (*right*) $\Delta \phi (\ell ,b)$ in (*top*) the CR1, (*middle*) the CR2, and (*bottom*) the CR3 control region, for the electron and muon channels combined. The distributions observed in data, depicted with the points, are compared with the predicted background contributions. In the CR2 and CR3 regions, the negligible multijet contribution is not shown, and neither is the $Z$+jets contribution in the CR3 region. The multijet background is normalised by the data-driven method, and the other backgrounds are normalised to their theoretical cross-sections. The error bands correspond to the uncertainties due to the statistical uncertainty of the sample added in quadrature with a conservative 50 % normalisation uncertainty on the multijet contribution, and with the $W$+jets and $t\bar{t}$ cross-section uncertainties. The ratios of the observed distributions to the predicted background distributions are shown in the lower frame

In order to validate the background model, three control regions orthogonal to the signal region are defined. Figure 3 is a sketch describing the signal and control regions in the ($m_{\mathrm {T}}(\ell , E_{\mathrm {T}}^{\mathrm {miss}})$, $|\Delta \phi (\ell ,b)|$)-plane. The first control region (CR1) is enriched in $W$+jets and multijet background events by requiring events to satisfy $60\mathrm {~GeV}<m_{\mathrm {T}}(\ell , E_{\mathrm {T}}^{\mathrm {miss}})<120\mathrm {~GeV}$ in addition to the pre-selection criteria. In the second control region (CR2) with a kinematic regime closer to the one of the signal region, the pre-selected events are required to satisfy $120\mathrm {~GeV}<m_{\mathrm {T}}(\ell , E_{\mathrm {T}}^{\mathrm {miss}})<150\mathrm {~GeV}$ and the azimuthal separation $\Delta \phi (\ell ,b)$ between the lepton and the $b$-tagged jet must be less than $1.8$. Finally, the third control region (CR3) is defined in order to validate the modelling of the background arising from $t\bar{t}$ events. An event sample dominated by $t\bar{t}$ events is obtained by selecting events with a second $b$-tagged jet; both $b$-tagged jets are identified with a $b$-tagging criterion with an efficiency of 80 %, the sub-leading jet satisfies $p_{\text {T}}<50$ GeV, and the events must satisfy $m_{\mathrm {T}}(\ell , E_{\mathrm {T}}^{\mathrm {miss}})>150\mathrm {~GeV}$ and $|\Delta \phi (\ell ,b)|<$ 1.8 in addition to the pre-selection criteria. The distributions of $m_{\mathrm {T}}(\ell , E_{\mathrm {T}}^{\mathrm {miss}})$ and of $\Delta \phi (\ell ,b)$ in the three control regions are depicted in Fig. 4. Reasonable agreement between the data and the predicted background estimate is found.

## Systematic uncertainties

The impact of systematic uncertainties is considered on the yields of individual background and signal processes. The main systematic uncertainties are those related to the jet energy scale, the $b$-tagging efficiency, the effect of the choice of PDF on signal and background acceptance, the effect of the choice of MC generator and of additional radiation on $t\bar{t}$ modelling, and the effect of the limited size of the samples.

### Sample size

Due to the stringent kinematic cuts in the signal regions, the impact of the limited size of the data and simulated samples on the signal and background estimates is a significant source of systematic uncertainty. For the $Z$+jets, multijet, and single-top-quark $s$- and $t$-channel processes, the expected event yield is zero in both channels, for the SRI and SRII selections, respectively. In such cases, a 68 % confidence level (CL) upper limit on the yields is calculated, assuming a Poisson distribution, and is taken into account in the limit-setting procedure. This upper limit represents at most 10 % of the background contribution.

For the other processes, which have non-negligible contributions, the effect of the limited sample size on expected signal (background) yields varies between 2 and 5 % (2 and 9 %).

### Object modelling

The effect of the uncertainty on the jet energy scale [67] is a change in the signal (background) event yields of 1–5 % (9–10 %), depending on the channel and on the signal region. The impact of the jet energy resolution uncertainty, evaluated by smearing the jet energy in the simulation [73], is a 2–3 % (1–2 %) effect on the signal (background) rates. The systematic uncertainty associated with the efficiency of the cut on the jet vertex fraction results in yield variations of 2–3 % (2–6 %) in the signal (background). Uncertainties on $b$-tagging efficiency and mistagging rates are estimated from data [68]; the effect on signal and background yields is 3–5 %. The jet reconstruction efficiency uncertainty has an effect below 1 %, except for the background in the SRII region (up to 3 %).

**Fig. 5:**
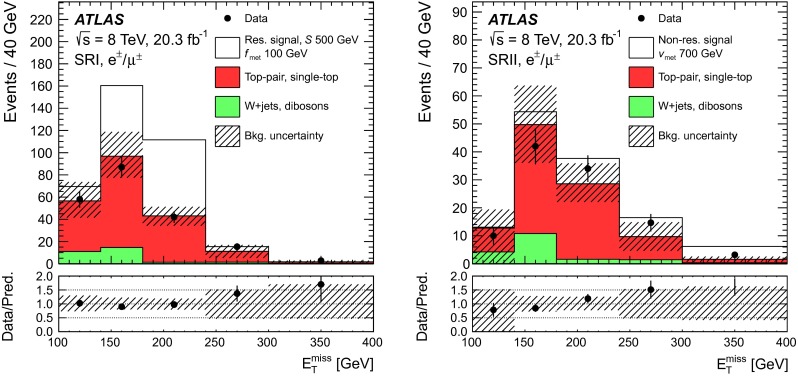
Distributions of $E_{\mathrm {T}}^{\mathrm {miss}}$ in the (*left*) SRI and (*right*) SRII signal regions, for the electron and muon channels combined. The distributions observed in data, depicted with the points, are compared with the predicted background contributions, shown stacked together with the expected resonant (non-resonant) signal contribution for the $m(f_{\mathrm {met}})=100$ GeV and $m(S)=500$ GeV ($m(v_{\mathrm {met}})=700$ GeV) hypothesis. The expected backgrounds are normalised to their theoretical cross-sections, and the expected resonant (non-resonant) signal is normalised to the theoretical cross-section corresponding to $a_{\mathrm {res}}=0.2$ ($a_{\mathrm {non\text{- }res}}=0.2$). The error bands on the expected backgrounds correspond to the uncertainties due to all systematic sources added in quadrature. The first (last) bin includes underflows (overflows). The ratios of the observed distributions to the predicted background distributions are shown in the lower frame

Smaller uncertainties arise from the lepton trigger, reconstruction, and identification efficiencies (up to 1 %) and from lepton energy scale and resolution (up to 1 % for signal and between 1 and 3 % for background). The systematic uncertainties related to leptons and jets are propagated to the $E_{\mathrm {T}}^{\mathrm {miss}}$. In addition, uncertainties on the estimation of the contributions of calorimeter energy deposits not associated with any reconstructed objects have an effect below 1 % (up to 4 %) on expected signal (background) contribution.

### Signal and background acceptance modelling

The uncertainties on the signal and background acceptance due to the choice of PDF are estimated using the CT10 [36, 37], MSTW2008 68 % CL NLO [32, 33] and NNPDF2.3 [49, 74] PDF sets with their uncertainties, following the PDF4LHC recommendations [48]. The variations of the signal (background) yields are between 4–11 % (5–6 %).

The dependence of the $t\bar{t}$ process on the generator and parton showering simulation is evaluated by comparing the nominal sample produced with Powheg+Pythia with three samples generated using the CT10 PDF, one sample produced with Powheg-Box v1_r2129, one sample using the Alpgen LO multileg generator v2.14 [53], and one sample produced using MC@NLO v4.06 [75, 76]. Herwig v6.52 [55] is used for parton showering and hadronisation and Jimmy v4.31 [77] for the underlying event. The largest variation, representing 5–11 % of the total background yield, arises from the comparison with the Alpgen+Herwig sample. For $Wt$ production, the nominal Powheg+Pythia sample is compared with a sample produced with MC@NLO v4.06, leading to a variation of 4–6 % on the total background yield. Furthermore, the uncertainty associated with the NLO calculation schemes for the $Wt$ process is evaluated by comparing the nominal sample generated with the diagram removal scheme to a sample using the diagram subtraction (DS) scheme [78]; this uncertainty is 3–5 % on the total background yield.

**Table 1:** Expected and observed event yields in the SRI (SRII) signal region, combining the electron and muon channels. The expected contribution of resonant (non-resonant) signal corresponding to the lowest and highest mass hypotheses considered in this analysis and of SM backgrounds are given. The first quoted uncertainty gives the uncertainty due to statistics. The second one gives the uncertainties due to all other systematic effects, symmetrised, regrouped, and summed quadratically, without taking into account possible anticorrelations between systematic uncertainties and between processes, for the purpose of this table

	SRI	SRII
Resonant signal, $m(S)=500$ GeV, $m(f_{\mathrm {met}})=0$ GeV	$253 \pm 5 \pm 34$	–
Resonant signal, $m(S)=500$ GeV, $m(f_{\mathrm {met}})=100$ GeV	$186 \pm 4 \pm 24$	–
Non-resonant signal, $m(v_{\mathrm {met}})=0$ GeV	–	$2{,}430 \pm 130 \pm 210$
Non-resonant signal, $m(v_{\mathrm {met}})=1{,}000$ GeV	–	$8.4 \pm 0.1 \pm 0.8$
$t\bar{t}$	$190 \pm 7 \pm 40$	$94 \pm 5 \pm 19$
Single-top $s$-channel	$<$0.05	$<$0.05
Single-top $t$-channel	$<$0.10	$<$0.10
Single-top $Wt$	$19 \pm 4 \pm 14$	$10 \pm 3 \pm 11$
$W$+light-quarks	$2 \pm 2 \pm 4$	$3 \pm 3 \pm 4$
$W$+$bb$	$10 \pm 3 \pm 5$	$9 \pm 3 \pm 7$
$W$+$cc$	$5 \pm 3 \pm 3$	$2 \pm 7 \pm 2$
$W$+$c$	$12 \pm 5 \pm 8$	$4 \pm 2 \pm 4$
Diboson	$1.3 \pm 0.6 \pm 0.7$	$1.0 \pm 0.5 \pm 0.5$
$Z$+jets	$<$4	$<$4
Multijet	$<$0.6	$<$1.3
Total background	$240 \pm 10$ $\pm 50$	$124 \pm 11$ $\pm 27$
Data	$238$	$133$

**Fig. 6:**
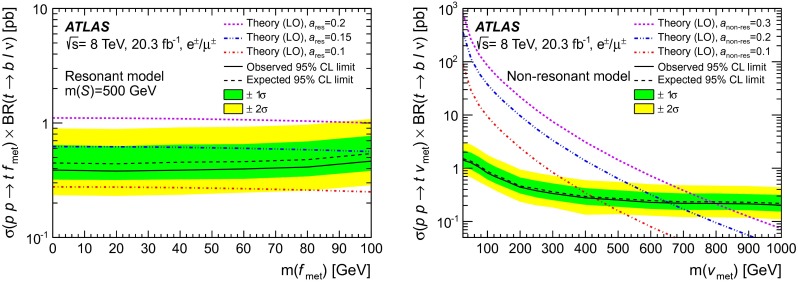
Observed and expected limits on the cross section times branching ratio (*left*) for the resonant model with $m(S)=500$ GeV and (*right*) for the non-resonant model, as a function of the mass of $f_{\mathrm {met}}$ and $v_{\mathrm {met}}$, respectively. The predicted signal cross-sections for different coupling strengths are also shown

The dependence of the $t\bar{t}$ event rate on additional radiation is evaluated using a $t\bar{t}$ sample generated with the AcerMC LO generator v3.8 [38, 39], with the CTEQ6L1 PDF set [40], and coupled with Pythia v6.426. The Pythia parameters are varied in a manner consistent with a measurement of $t\bar{t}$ production with additional jet activity [79]. The related variation in the total background is around 5 % (9 %) in the SRI (SRII) region.

### Background normalisation

Theoretical uncertainties are $-$5.9/+5.1 % for the inclusive $t\bar{t}$ cross-section [41–47], and 6.8 % for the $Wt$-channel cross-section [51]. An uncertainty of 24.5 % on diboson and $W$+light-quarks rates is also assigned. These estimates come from the uncertainty on the inclusive diboson and $W$-boson production cross-sections [57] (5 and 4 %, respectively) and from a conservative assessment based on a prediction for the ratio of the event rate with $n+1$ jets to the event rate with $n$ jets [80, 81], resulting in 24 % per additional jet, added in quadrature. A 50 % uncertainty, as evaluated in Ref. [82], is assigned to the $W$+$bb$, $W$+$cc$, and $W$+$c$ rates.

### Luminosity

The uncertainty on the integrated luminosity is 2.8 % [25], affecting the signal estimates as well as the simulated backgrounds.

## Results and interpretation

Figure 5 shows the distributions of $E_{\mathrm {T}}^{\mathrm {miss}}$ in the SRI and SRII signal regions, comparing the data to the expected signal and background contributions. The expected resonant (non-resonant) signal contribution for the $m(f_{\mathrm {met}})=100$ GeV ($m(v_{\mathrm {met}})=700$ GeV) hypothesis, normalised to the theoretical cross-section corresponding to $a_{\mathrm {res}}=0.2$ ($a_{\mathrm {non\text{- }res}}=0.2$), is also shown.

Table 1 reports the expected event yields for the background and signal processes and the observed event yields in the SRI and SRII signal regions. As no excess is observed in data, 95 % CL upper limits on the signal production cross-sections are set with the $\mathrm{CL}_s$ procedure [83, 84]. A log-likelihood ratio (LLR) is used as the test statistic, defined as the ratio of the signal-plus-background hypothesis to the background-only hypothesis. For a given hypothesis, the combined likelihood is the product of the likelihoods for the two channels considered (electron and muon), each resulting from the product of a Poisson distribution representing the statistical fluctuations of the expected total event yield, and of Gaussian distributions representing the effect of the systematic uncertainties. Pseudo-experiments are generated for both hypotheses, taking into account correlations across channels and processes. The fraction of pseudo-experiments for the signal-plus-background (background-only) hypothesis with LLR larger than a threshold defines $\mathrm{CL}_{s+b}$ ($\mathrm{CL}_{b}$). This threshold is set to the observed (background median) LLR for the observed (expected) limit. Signal cross-sections for which $\mathrm{CL}_s = \mathrm{CL}_{s+b}/\mathrm{CL}_b < 0.05$ are considered excluded at the 95 % CL.

**Fig. 7:**
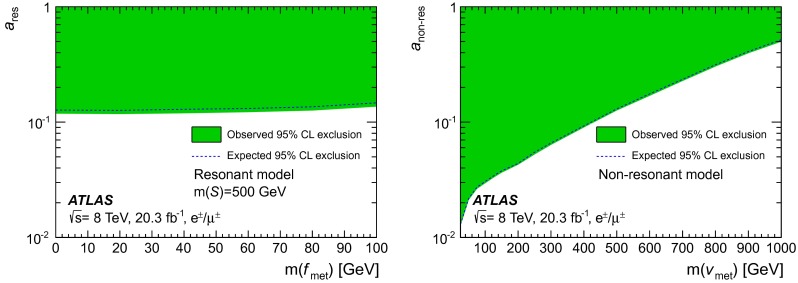
Observed and expected excluded coupling strengths (*left*) for the resonant model with $m(S)=500$ GeV and (*right*) for the non-resonant model, as a function of the mass of $f_{\mathrm {met}}$ and $v_{\mathrm {met}}$, respectively

Figure 6 shows the expected and observed 95 % CL excluded cross-section times branching ratio as a function of the mass of the invisible state, for each of the two signal models. In the case of the resonant model, cross-sections corresponding to an effective coupling strength $a_{\mathrm {res}}=0.2$ are excluded in the whole mass range, but not cross-sections corresponding to $a_{\mathrm {res}}=0.1$. For the non-resonant model, cross-sections corresponding to $a_{\mathrm {non\text{- }res}}=0.1$ ($0.2$, $0.3$) are excluded up to $m(v_{\mathrm {met}})=432$ GeV ($657$ GeV, $796$ GeV).

**Table 2:** Expected and observed 95 % CL limits on the effective coupling $a_{\mathrm {res}}$ as a function of the mass of the invisible state for the resonant model

$m(f_{\mathrm {met}})$ (GeV)	95 % CL upper limit on $a_{\mathrm {res}}$	
	Expected	Observed
0	0.13	0.12
20	0.13	0.12
40	0.13	0.12
60	0.13	0.12
80	0.14	0.13
100	0.15	0.14

**Table 3:** Expected and observed 95 % CL limits on the effective coupling $a_{\mathrm {non\text{- }res}}$ as a function of the mass of the invisible state for the non-resonant model

$m(v_{\mathrm {met}})$ (GeV)	95 % CL upper limit on $a_{\mathrm {non\text{- }res}}$	
	Expected	Observed
0	0.03	0.029
25	0.013	0.013
50	0.022	0.021
75	0.027	0.026
100	0.031	0.030
125	0.034	0.033
150	0.038	0.036
200	0.044	0.043
250	0.055	0.052
300	0.066	0.063
400	0.093	0.090
500	0.13	0.13
600	0.18	0.17
700	0.24	0.23
800	0.32	0.30
900	0.41	0.40
1,000	0.52	0.50

The cross-sections are proportional to the square of the effective coupling. Thus, a 95 % CL upper limit on $a_{\mathrm {res}}$ and $a_{\mathrm {non\text{- }res}}$ as a function of the mass of the invisible states is extracted. The results are shown in Fig. 7. This upper limit is set assuming that the coupling has no effect on the signal acceptance modelling. In the case of the resonant model, in which the increase of the resonance width with increasing coupling strength changes the signal kinematics, this assumption is validated by using two dedicated simulated samples produced with $a_{\mathrm {res}}=0.5$ and $a_{\mathrm {res}}=1.0$ instead of $a_{\mathrm {res}}=0.2$. These two hypotheses are excluded at 95 % CL with the same limit-setting procedure. Since the kinematic distributions are similar in the whole $m(f_{\mathrm {met}})$ range, this assumption is valid for all values of the $f_{\mathrm {met}}$ mass. Tables 2 and 3 give the expected and observed 95 % CL upper limits on the effective coupling as a function of the mass of the invisible state, for the resonant and non-resonant model, respectively.

## Summary and conclusion

Monotop events are searched for in the $\sqrt{s}=\mathrm {8~TeV}$ $pp$ collision data collected in 2012 by the ATLAS experiment at the LHC corresponding to an integrated luminosity of 20.3 $\mathrm {fb}^{-1}$. Two classes of signal models are studied, producing right-handed top quarks together with exotic neutral particles giving rise to missing energy. The semi-leptonic decay mode of the top quark is exploited: events with one isolated electron or muon and one $b$-tagged jet are selected. No significant deviation from the standard model predictions is observed. Upper limits on the signal cross-sections and on the corresponding effective couplings are set at 95 % CL using the $\mathrm{CL}_s$ method. In the case of the production of a 500 GeV spin-0 resonance, effective coupling strengths above $a_{\mathrm {res}}=0.15$ are excluded for a mass of the invisible spin-1/2 state between 0 and 100 GeV. In the case of non-resonant production, effective coupling strengths above $a_{\mathrm {non\text{- }res}}=0.1$, 0.2, and 0.3 are excluded for a mass of the invisible spin-1 state up to 432, 657, and 796 GeV, respectively. The observed 95 % CL limits are compatible with the expectations.

## Footnotes

1 The ATLAS experiment uses a right-handed coordinate system with its origin at the nominal interaction point (IP) in the centre of the detector and the $z$-axis along the beam pipe. The $x$-axis points from the IP to the centre of the LHC ring, and the $y$-axis points upwards. Cylindrical coordinates $(r,\phi )$ are used in the transverse plane, $\phi$ being the azimuthal angle around the beam pipe. The pseudorapidity is defined in terms of the polar angle $\theta$ as $\eta =-\ln \tan (\theta /2)$.

2 Extra proton–proton interactions from the same and previous bunch-crossings.

3 The transverse mass is defined as $m_{\mathrm {T}}(\ell , E_{\mathrm {T}}^{\mathrm {miss}})= \sqrt{2 p_{\mathrm {T}}\left( \ell \right) E_{\mathrm {T}}^{\mathrm {miss}}\left( 1-\cos \Delta \phi \left( p_{\mathrm {T}}\left( \ell \right) , E_{\mathrm {T}}^{\mathrm {miss}}\right) \right) }$, where $p_{\mathrm {T}}\left( \ell \right)$ denotes the modulus of the lepton transverse momentum, and $\Delta \phi \left( p_{\mathrm {T}}\left( \ell \right) , E_{\mathrm {T}}^{\mathrm {miss}}\right)$ the azimuthal difference between the missing transverse momentum and the lepton directions.
